# Mathematical Modeling of Population Dynamics of Pollinators: A Survey

**DOI:** 10.3390/biology14091308

**Published:** 2025-09-22

**Authors:** Fernando Huancas, Anibal Coronel, Esperanza Lozada, Jorge Torres

**Affiliations:** 1 Departamento de Matemática, Facultad de Ciencias Naturales, Matemáticas y del Medio Ambiente, Universidad Tecnológica Metropolitana, Ñuñoa, Santiago 7750000, Chile; 2 Departamento de Ciencias Básicas, Facultad de Ciencias, Universidad del Bío-Bío, Campus Fernando May, Chillán 3780000, Chile; elozada@ubiobio.cl (E.L.); jotorres@ubiobio.cl (J.T.)

**Keywords:** pollinators, plant–pollinator interaction, pesticides

## Abstract

In this paper, we develop a systematic review of the existing literature on the mathematical modeling of several aspects of pollinators. We selected the MathSciNet and Wos databases and performed a search for the words “pollinator” and “mathematical model”. This search yielded a total of 236 records. After a detailed screening process, we retained 107 publications deemed most relevant to the topic of mathematical modeling in pollinator systems. We conducted a bibliometric analysis and categorized the studies based on the mathematical approaches used as the central tool in the mathematical modeling and analysis. The mathematical theories used to obtain the mathematical models were ordinary differential equations, partial differential equations, graph theory, difference equations, delay differential equations, stochastic equations, numerical methods, and other types of theories, like fractional order differential equations. Meanwhile, the topics were positive bounded solutions, equilibrium and stability analysis, bifurcation analysis, optimal control, and numerical analysis. We summarized the research findings and identified some challenges that could inform the direction of future research, highlighting areas that will aid in the development of future research.

## Simple Summary

Pollinators are crucial for diverse biological processes, but it is recognized that there has been a decline in their populations in recent years. Hence, the study of the population dynamics of pollinators is a relevant topic for research. In this study, to contribute to the state of the art of mathematical modeling of population dynamics, we searched the relevant literature in two databases. This review explores the different contributions, develops a summary and classification, and states some future work to understand the behavior of pollinator interactions.

## 1. Introduction

In the last few decades, the study of pollinators has attracted the attention of several researchers, as it is known that pollinators play a crucial role as ecosystem regulators in nature [1,2,3,4,5]. It is known that there are several types of pollinators, including birds, bats, butterflies, moths, flies, beetles, wasps, small mammals, and, most importantly, insects like bees. These animals are responsible for the bulk of pollination, which significantly affects our daily lives. Some important facts about pollinators are that three out of four crops depend on pollinators; in the extreme case of total disappearance of pollinators, this would lead to a decrease in world food production; and the causes of pollinator decline include disease, climate change, and pesticides. The problem associated with pollinators is complex and should be analyzed from multiple scientific perspectives, particularly biology, chemistry, and mathematics.

Pollination is a crucial event in the reproductive cycle of flowering plants. In this context, several characteristics associated with the evolutionary process of species help maintain and optimize the functioning of various ecosystems [6]. Two widely studied phenomena are flowers that produce nectar and those that do not. First, we consider flowers that produce nectar to be a food source. Some plants provide nectar to pollinators as a reward for their assistance in pollination. In this context, a notable aspect is the fact that plants conceal their nectar, which prevents pollinators from detecting its presence without first entering the flower. Second, we know that there are plants that do not produce nectar. Nectar production requires a considerable amount of energy; some flowers can employ deceptive strategies by not producing nectar. Despite lacking nectar, these flowers can still be pollinated by pollinators. An example of this type of plant is found in certain orchid species, which are pollinated through a phenomenon known as Batesian mimicry. Nectarless flowers are likely the result of evolutionary optimization. Additionally, other pollination-related phenomena include the fact that flowers can also attract pollinators by producing large floral displays, even if they provide no reward. There are pollinators skilled at extracting nectar without pollinating the flower, known as nectar robbers. There are also indirect pollinators, such as ants, which seek other plant nutrients or prey on insects living on the plant, rather than directly seeking nectar or pollen.

In this paper, we aim to elucidate the existing studies on pollinators from a mathematical perspective. Several phenomena related to pollinators can be analyzed using mathematical modeling, such as the dynamics of pollinator populations, plant–pollinator interactions, the effects of climate change on pollinator decline, the impact of pesticides on pollinator populations, and the spread of infectious diseases among pollinators. A recent review developed by Chen et al. introduced the framework of different mathematical models related to the dynamics of honeybee populations [7]. However, to the extent of our knowledge, there is no comprehensive review of the state of the art in mathematical modeling of pollinators and related topics. Therefore, we conducted a systematic literature review using bibliometric methods and following the methodology detailed in [8] (see also [9]).

We surveyed the MathSciNet and WoS databases and examined the topics of each work. We obtained a set of 107 works, comprising 105 journal articles, 1 PhD thesis, and 1 book chapter related to the mathematical modeling of pollinators. The retained list of articles ranged from 1978 to 2025. We analyzed the papers and established a classification based on the mathematical theory involved in the mathematical modeling formulation. The classification introduced considers four groups: ordinary, differential equation models, partial differential equation models, network-based models, and other methodologies. In the case of other methodologies, we found discrete mathematical-based models, stochastic models, and others. We outlined some key contributions of the papers and compiled a list of topics that highlight potential challenges and perspectives for further research on the topic.

This paper is outlined as follows. In Section 2, we describe the methodology, including the list of selected relevant works that were identified and analyzed, as well as the bibliometric analysis. In Section 3, we report the results of the main findings arising from analysis of the existing literature on the mathematical modeling of pollinators. In Section 4, we discuss some biological issues of the retained list. In Section 5, we collect some aspects which are not included in the previous sections but are relevant for the completeness of the work. Finally, in Section 6, we present the conclusions of the paper and also outline some possible future research directions.

## 2. Methodological Framework

**Figure 1:**
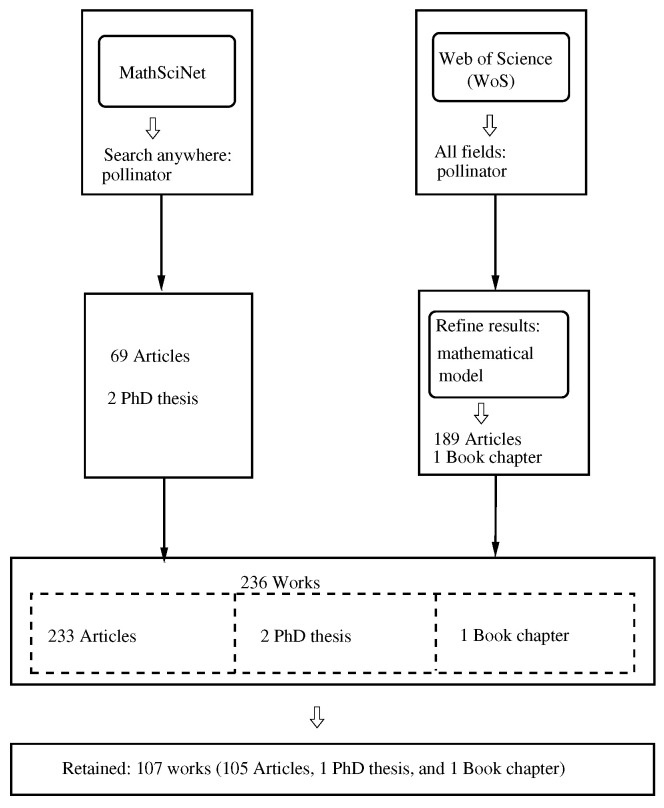
Schematic summary of the process used for identifying the relevant work (see Section 2).

The methodology supporting the present work combines two approaches to developing a literature review: a systematic review and a bibliometric analysis. To be more precise, we adopted the methodology presented in [8], which consists of the five steps given in [9]: (1) framing questions for a review, (2) identifying relevant work, (3) assessing the quality of studies, (4) summarizing the evidence, and (5) interpreting the findings. The results for steps (1) and (2), step (3), and steps (4) and (5) are presented below in Section 2.1, Section 2.2, and Section 3, respectively. A synthesized visualization is presented in Figure 1.

### 2.1. Framing Questions for a Review and Identifying Relevant Work

We considered the following two questions:

Question 1: What are the studies developed for mathematical modeling of the pollinator population’s dynamics?
Question 2: What types of modeling approaches were used in those studies?
Meanwhile, related to the step of identification of the relevant work, we selected two databases, MathSciNet and the Web of Science (WoS), with the following details:

- *MathSciNet* (https://mathscinet.ams.org/mathscinet/, accessed on 14 April 2025): We searched for the word “pollinator” using the option “search term: anywhere” and found that the response reported a total of 71 items: 69 journal articles and 2 PhD theses.
- *WoS* (https://www.webofscience.com/, accessed on 14 April 2025). We used the option “all fields” for the platform’s search engine to search for the word “pollinator”, obtaining 26,938 items. Then, by using the keyword “mathematical model” in the option “refine results”, we found 199 items: 198 journal articles and 1 book chapter.

When combining the two lists, we found that there were 34 duplicated items. Then, we obtained a list of 236 works: 233 journal articles, 2 PhD theses, and 1 book chapter. Here, we note that the search in both databases was not limited to the keyword “pollinator” being specified in the works.

We performed an examination of the 236 works and retained those which were related, namely with mathematical modeling as the topic of the paper, obtaining a list of 107 works [10,11,12,13,14,15,16,17,18,19,20,21,22,23,24,25,26,27,28,29,30,31,32,33,34,35,36,37,38,39,40,41,42,43,44,45,46,47,48,49,50,51,52,53,54,55,56,57,58,59,60,61,62,63,64,65,66,67,68,69,70,71,72,73,74,75,76,77,78,79,80,81,82,83,84,85,86,87,88,89,90,91,92,93,94,95,96,97,98,99,100,101,102,103,104,105,106,107,108,109,110,111,112,113,114,115,116]. In [19], there is a book chapter in conference proceedings, while [31] is a PhD thesis, and the other works are journal articles. We note two additional facts: the present review is registered in OSF (https://doi.org/10.17605/OSF.IO/3DWSR, accessed on 5 September 2025)), and for the inclusion criteria, we considered a work to be about mathematical modeling of pollinators when there was a proposal to research the population dynamics of pollinators.

### 2.2. Assessing the Quality of the Studies

**Figure 2:**
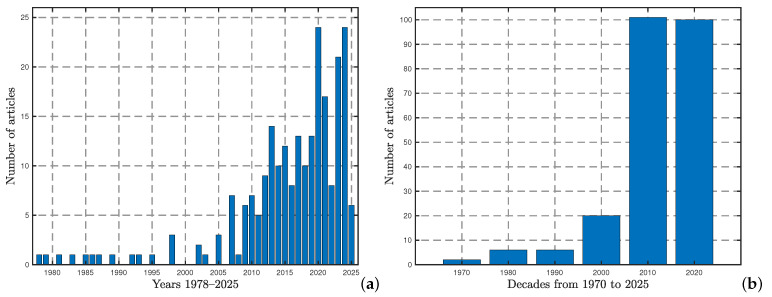
Number of works in MathSciNet and the WoS related to the keywords “pollinators” and “mathematical models”. (**a**) Number of articles by year from 1978 to 2025. (**b**) Number of articles by decade.

**Figure 3:**
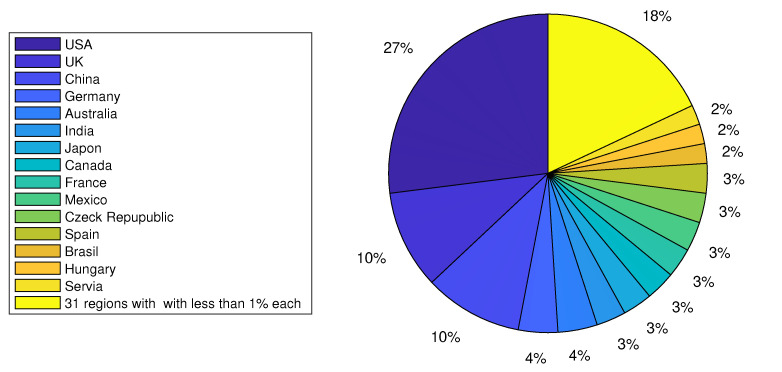
Percentages of the number of authors according to the geographic locations declared by the authors. We rounded off all percentages to their integers.

A graphical distribution of the list, by year and by decade from 1978 to 2025, is shown in Figure 2. Here, we observe that the oldest reference was from 1978, and the most recent one was from 2025. Also, we noticed a clear increase in articles over the decades, even though there were slight decreases in some years. Additionally, we also noted the geographic location declared by each of the authors in the corresponding affiliation for each article; the results are graphically presented in Figure 3. The affiliations of the authors were counted in the 236 works. The details for the retained list are given in Appendix A and more specifically in Table A1. The regions with the highest number of records were the United States of America (USA), the United Kingdom (UK), and China, with 205, 102, and 100 records, corresponding to 27%, 10%, and 10% of the works, respectively. These rankings were followed by regions with less than 4% representation as detailed below:

- Brazil (45) and Australia (35) with 4% each;
- India (29), Japan (29), Canada (28), France (28), Mexico (28), the Czech Republic (26), and Spain (25) with 3% each;
- Brazil (22), Hungary (21), and Serbia (16) with 2% each;
- Italy (14), South Africa (13), Denmark (11), Israel (10), Sweden (10), Taiwan (10), the Netherlands (9), New Zealand (9), Chile (8), Russia (8), Norway (7), Argentina (6), and Belgium (5) with 1% each;
- Bulgaria (4), Finland (4), Poland (4), the Republic of Korea (4), Switzerland (4), the Philippines (3), Slovakia (3), Greece (2), Kenya (2), Estonia (2), Ecuador (1), Indonesia (1), Ireland (1), Pakistan (1), Portugal (1), Saudi Arabia (1), Slovenia (1), and Thailand (1) with 0% each.

Here, the number in parentheses is the number of records for the region. A graphical interpretation is given in Figure 3, with The oldest article being [5].

The indicators for journals and authors in the list are presented below. The retained articles in the list were published in 59 journals. Table 1 shows the nine journals which were in the first four positions according the published articles. We found that there were 13 journals with 2 publications and 37 journals with 1 publication. In Table 2, we show the top 10 journals according to the H index of the SCImago Journal & Country Rank (https://www.scimagojr.com/ accessed on 4 May 2025), and the SJR 2023 indicators, quartiles, and subject areas of those journals were obtained from SJR (https://jcr.clarivate.com/ accessed on 4 May 2025). Refer to Appendix B for more details. Moreover, in Table 3, we present the top four prolific authors. Meanwhile, Table 4 and Table 5 provide an extensive and structured synthesis of the retained literature. In Table 4, we detail the main findings of [10,11,12,13,14,15,16,17,18,19,20,21,22,23,24,25,26,27,28,29,30,31,32,33,34,35,36,37,38,39,40,41,42,43,44,45,46,47,48,49,50,51,52,53,54,55,56,57,58,59,60,61,62,63,64,65,66,67,68,69,70,71,72,73,74,75,76,77,78,79,80,81,82,83,84,85,86,87,88,89,90,91,92,93,94,95,96,97,98,99,100,101,102,103,104,105,106,107,108,109,110,111,112,113,114,115,116], and in Table 5, we organized the results by the seven major modeling domains identified (see Section 3.5). Each domain is characterized by its underlying biological assumptions, mathematical formulation, parametrization strategies, validation or calibration procedures, and key ecological insights. The table also highlights the potential policy implications derived from each modeling approach, thereby bridging theoretical contributions with applied relevance. This typological classification facilitates comparative analysis across studies and supports interdisciplinary integration of mathematical ecology, conservation planning, and empirical calibration. Representative references are included to exemplify each category and guide further exploration of methodological trends and thematic priorities.

## 3. Summarizing the Evidence and Interpreting the Findings

In Section 2.1, we introduced two questions. “Question 1” is clearly answered by the retained list [10,11,12,13,14,15,16,17,18,19,20,21,22,23,24,25,26,27,28,29,30,31,32,33,34,35,36,37,38,39,40,41,42,43,44,45,46,47,48,49,50,51,52,53,54,55,56,57,58,59,60,61,62,63,64,65,66,67,68,69,70,71,72,73,74,75,76,77,78,79,80,81,82,83,84,85,86,87,88,89,90,91,92,93,94,95,96,97,98,99,100,101,102,103,104,105,106,107,108,109,110,111,112,113,114,115,116]. Meanwhile, to answer “Question 1”, we introduce two classifications pertaining to the mathematical approaches used and the topics researched. We revised the retained list and defined the following four groups according to the mathematical theories applied for mathematical modeling:

(1) Ordinary differential equations group (see Section 3.1): Here, we distinguish between three types of models based on the methodology used for modeling. First, we considered the Lotka–Volterra models for two populations (see Section 3.1.3). The works of this type are [10,11,15,22,28,30,33,47,55,68,73,76,86,88,91,96,104,106]. The second type was the Lotka–Volterra models for more than two populations (see Section 3.1.2), with the articles being [15,16,19,24,28,30,33,34,37,42,44,45,46,48,51,62,63,64,67,70,71,74,75,78,79,81,87,100,107,114,115]. The third class of ordinary differential equations systems was obtained via application of the compartmental methodology (see Section 3.1.3), and the works of this type are [14,36,38,39,41,61,83,85,89,90,93,98,105,110].
(2) Partial differential equations group (see Section 3.1): The generalization of the ordinary differential equations to include the spatial displacement of pollinators was studied in [19,26,27,31,32,50,111].
(3) Patch network (see Section 3.3): Consideration of groups of pollinators in different patches and the interaction of network concepts was conducted in [13,21,25,35,40,43,49,52,54,65,69,80,82,95,97,99,101,103,108,113]
(4) Other methodologies (see Section 3.4): Other kinds of works like letters, reviews, and emergent methodologies like fractional-order or delay models were introduced in [12,17,18,20,23,26,29,53,56,57,58,59,60,66,84,94,102,104,109].

Extensive details are presented below in Section 3.1, Section 3.2, Section 3.3 and Section 3.4. We include a summarization of the topics covered by the studies in Section 3.5. We note that some works were considered to be in more than one group, as they examined mathematical modeling from multiple perspectives. For instance, the more typical case is that partial differential equation models are obtained as a generalization of ordinary differential equation models, and the same work can be considered to be in groups (1) and (2) (see, for example, [19]). Another example is [110], which involves multiple species and a class-structured (compartmental) framework, and it also incorporates nonlinear interaction terms that are characteristic of Lotka–Volterra systems. Therefore, it could alternatively be included in Section 3.1.2. However, it was placed in this subsection because the study’s primary focus lies in disease propagation and infestation dynamics rather than population-level coexistence or competitive interactions. This aligns it more closely with classical compartmental models such as SIR, SEIR, and related formulations. Furthermore, we obtained a Cohen’s kappa coefficient of 0.78.

### 3.1. Mathematical Models Based on Ordinary Differential Equations

#### 3.1.1. Two Population Mathematical Models Using Lotka–Volterra-Like Methodologies

In the retained list, there are several works where the mathematical models were obtained through the interaction of two populations, such as plants and pollinators. The two basic assumptions required to obtain these mathematical models were as follows: there is an interaction between the pollinator and the plant, and the impact of various ecological and environmental variables is neglected. Then, the modeling approach focused on how the birth and death rates of both populations drove their changes or, equivalently, how the birth, death, and interactions of the populations affected population growth. Then, we deduced that

(1) $$\begin{array}{cc} \left[\begin{array}{c} \mathrm{rate}\;\mathrm{of}\;\mathrm{change}\;\mathrm{of}\\ \mathrm{plants}\\ \mathrm{population} \end{array}\right] & =\left[\begin{array}{c} \mathrm{plants}\\ \mathrm{population}\\ \mathrm{birth}\;\mathrm{rate} \end{array}\right]-\left[\begin{array}{c} \mathrm{plants}\\ \mathrm{population}\\ \mathrm{dead}\;\mathrm{rate} \end{array}\right], \end{array}$$

(2) $$\begin{array}{cc} \left[\begin{array}{c} \mathrm{rate}\;\mathrm{of}\;\mathrm{change}\;\mathrm{of}\\ pollinator\\ population \end{array}\right] & =\left[\begin{array}{c} pollinator\\ population\\ birth\;rate \end{array}\right]-\left[\begin{array}{c} pollinator\\ population\\ dead\;rate \end{array}\right]. \end{array}$$

Let us denote the total population of plants and pollinators as *p* and *a*, respectively. Then, we have

(3) $$\begin{array}{c} \left[\begin{array}{c} rate\;of\;change\;of\\ plants\\ population \end{array}\right]=\frac{dp}{dt},\;\left[\begin{array}{c} rate\;of\;change\;of\\ plants\\ population \end{array}\right]=\frac{da}{dt}\cdot \end{array}$$

To model the birth and death rates, we must consider several assumptions. To specify the algebraic forms modeling birth and death rates, we considered the discussion provided in [10]. We note that they modeled the plant–pollinator interaction via analogy with the Lotka–Volterra or prey-predator systems, assuming that the plants are predators and the pollinators are the prey.

The deduction of plant and pollinator birth rates in [10] was obtained by considering the following two assumptions: the plants are self-incompatible, and the plant birth rate is related to flower visits by the pollinators. They considered that the birth rate is proportional to the pollinator visits, neglected some factor like the finite supply of ovules, and assumed that the number of pollinator visits was modeled by a Holling’s functional such that the model was of the following form:

(4) $$\begin{array}{c} \left[\begin{array}{c} plants\\ population\\ birth\;rate \end{array}\right]=k_{1}a\left[\begin{array}{c} pollinator\\ plant\\ visit\;rate \end{array}\right]=k_{1}a\frac{\alpha p}{1+\alpha \beta p}, \end{array}$$

where $k_{1},\alpha$, and β model the number of ovules fertilized per visit, the searching rate constant multiplied by the encounter probability, and the handling time per visit, respectively. The encounter probability and the handling time depend on the energetic reward. More precisely, we have

(5) $$\begin{array}{c} \alpha =\sigma \mu ,\;\beta =\phi \mu , \end{array}$$

where σ is the probability of an encounter, ϕ is the reciprocal speed of nectar extraction, and μ is the energetic reward. By combining Equation (5) with Equation (4), we deduce that

(6) $$\begin{array}{c} \left[\begin{array}{c} plants\\ population\\ birth\;rate \end{array}\right]=\frac{k_{1}\sigma \mu ap}{1+\sigma \phi \mu^{2}p}\cdot \end{array}$$

It is assumed that the pollinator birth depends on the density and some variables such as the competition for nest sites or protein resources such that

(7) $$\begin{array}{c} \left[\begin{array}{c} pollinator\\ population\\ birth\;rate \end{array}\right]=a\left(\delta -\epsilon a\right), \end{array}$$

where δ is the maximum per capita pollinator birth rate and ϵ is the density-dependent regulation constant.

The deduction of plant and pollinator dead rates introduced in [10] is as follows. In the case of plant mortality, assume that it is proportional to the plant density, i.e., we have

(8) $$\begin{array}{c} \left[\begin{array}{c} plant\\ population\\ dead\;rate \end{array}\right]=\gamma p, \end{array}$$

where γ is the mortality rate. Meanwhile, for pollinators, it is assumed that the mortality pollinator rate is inversely related to the rate of energy intake, which in turn is jointly proportional to the visit rate and the energetic reward:

(9) $$\begin{array}{c} \left[\begin{array}{c} pollinator\\ population\\ dead\;rate \end{array}\right]=\lambda a-k_{2}a\left[\begin{array}{c} pollinator\\ plant\\ visit\;rate \end{array}\right]\mu =\lambda a-\frac{k_{2}\sigma \mu^{2}ap}{1+\sigma \phi \mu^{2}p}, \end{array}$$

where $k_{2}$ is a constant of energetic transformation and λ is the maximum death rate of pollinators in the absence of plants.

By combining Equations (3), (6)–(9) with Equations (1) and (2), we obtain a system of the form

(10) $$\begin{array}{c} \frac{dx_{1}}{dt}=f\left(x_{1},x_{2}\right)x_{1},\;\frac{dx_{2}}{dt}=g\left(x_{1},x_{2}\right)x_{2}, \end{array}$$

where

(11) $$\begin{array}{c} \left(x_{1},x_{2}\right)=\left(p,a\right),\;f\left(p,a\right)=\frac{k_{1}\sigma \mu a}{1+\sigma \phi \mu^{2}p}-\gamma ,\;g\left(p,a\right)=\epsilon \left(\frac{\delta -\lambda }{\epsilon }-a\right)+\frac{k_{2}\sigma \mu^{2}p}{1+\sigma \phi \mu^{2}p}\cdot \end{array}$$

Here, we observe that (δ−λ)/ϵ is the carrying capacity of the pollinator population. Other mathematical models of the general form in Equation (10) were introduced in [11,15,22,28,30,33,47,55,68,73,76,86,88,91,96,104,106]. We remark that there are eight types of models, depending on the interaction populations: plant–pollinator [10,11,15,22,28,30,55,68,73,91,96], plant–robber [28], pollinator–secretor [33], pollinator–cheater [33], plant–novice pollinator [47], novice pollinator–expert pollinator [47], plant–plant [76,86], juvenile pollinator–adult pollinator [88,104], and honeybee–mite [106].

#### 3.1.2. More Than Two Population Mathematical Models Using Lotka–Volterra-Like Methodologies

Analogous to the analysis developed in Section 3.1.1, we can consider that more than two species are interacting. To illustrate the concept, we consider the model introduced in [15], where the authors examined the interaction among three species—herbivores, plants, and pollinators—with populations denoted by *x*, *y*, and *z*, respectively. Then, by realizing a balance of birth rates, death rates, and interaction of the three populations, they deduced that the mathematical model is given by

(12) $$\begin{array}{cc} \frac{dx}{dt} & =bx\left(K-x\right)+\frac{g\left(z\right)k_{2}\mu^{2}\sigma xy}{1+\phi \sigma \mu^{2}y}, \end{array}$$

(13) $$\begin{array}{cc} \frac{dy}{dt} & =\frac{g\left(z\right)k_{1}\mu \sigma xy}{1+\phi \sigma \mu^{2}y}-\gamma y-\frac{m_{1}yz}{a+y}, \end{array}$$

(14) $$\begin{array}{cc} \frac{dz}{dt} & =\frac{m_{2}yz}{a+y}-\delta z, \end{array}$$

where *b* is a density-dependent regulation constant, *K* measures the diversity of pollinators of plants, $k_{2}$ is a constant of energetic transformation, μ is the energetic reward, σ is the probability of an encounter, ϕ is the reciprocal speed of nectar extraction, $k_{1}$ is an efficiency constant representing the number of ovules fertilized per visit, γ is the plant mortality rate, δ is the pollinator mortality rate, *a* is the half-saturation constant, $m_{1}$ is the maximal ingestion rate, $m_{2}$ is the herbivore maximal growth rate, and g(z) is a function depending on the herbivore population density. We observe that Equations (12)–(14) can be rewritten in the following general form:

(15) $$\begin{array}{c} \frac{dx_{i}}{dt}=f_{i}\left(x_{1},\ldots ,x_{d}\right)x_{i},\;i=1,\ldots ,d, \end{array}$$

where $x_{i}$ is the density of the *i*th species, *d* is the number of interacting species, and $f_{i}$ models the birth rates, death rates, and interactions.

The mathematical models of the general form in Equation (10) with d≥3 were introduced in the following 31 works from the retained list: [15,16,19,24,28,30,33,34,37,42,44,45,46,48,51,62,63,64,67,70,71,74,75,78,79,81,87,100,107,114,115]. In these works, the interacting populations were described as follows: plant–pollinator–hervibore [15,19,24,42,67,74,107], plant–pollinator–ant [24,44,45,46,51], plant–pollinator–robber [28,30,34,37,48,64], pollinator–secretor–cheater [33], plant–pollinator–flower [70,71,78,87], plant–novice pollinator-expert pollinator [47], two plants and one pollinator [63], two plants and two pollinators [62], plant–pollinator–pesticide [75], plant–pollinator–predator [100], plant–pollinator– parasite [114,115], plant–pollinator–green house gases–temperature [79], plant–seed–pollinator–seed disperser [81], and three general species [16].

#### 3.1.3. Mathematical Models Based on Compartmental Methodology

The pollination system is formed by plants and pollinators. The plant population is divided into three sub-populations: susceptible ($x_{v}$), pollinated ($x_{f}$), and infected by a fungus ($x_{i}$). Similarly, the pollinator population is divided into three states: can carry neither pollen nor fungal spores ($y_{n}$), can carry pollen ($y_{p}$), and can carry spores ($y_{sp}$). Moreover, we consider that the pollinated plants have a rate of return to the susceptible class. Then, by considering other assumptions regarding the interaction, Ingvarsson and Lundberg [14] introduced the following mathematical model:

$$\begin{array}{cc} \frac{dx_{v}}{dt} & =\left(b+\delta \right)x_{f}-\frac{\omega \beta_{1}y_{p}x_{v}}{x_{v}+x_{f}+x_{i}}-\frac{\omega \beta_{2}y_{sp}x_{v}}{x_{v}+x_{f}+x_{i}}-\mu x_{v},\\ \frac{dx_{f}}{dt} & =\frac{\omega \beta_{1}y_{p}x_{v}}{x_{v}+x_{f}+x_{i}}-\left(\mu +\delta \right)x_{f},\\ \frac{dx_{i}}{dt} & =\frac{\omega \beta_{2}y_{sp}x_{v}}{x_{v}+x_{f}+x_{i}}-\mu x_{i},\\ \frac{dy_{n}}{dt} & =kN-\nu \beta_{3}x_{v}\frac{y_{n}}{N}-\nu \beta_{4}x_{i}\frac{y_{n}}{N}-\gamma \left(y_{p}+y_{sp}\right)-ky_{n},\\ \frac{dy_{p}}{dt} & =\nu \beta_{3}x_{v}\frac{y_{n}}{N}-\nu \beta_{4}x_{i}\frac{y_{n}}{N}-\left(\gamma +k\right)y_{p},\\ \frac{dy_{sp}}{dt} & =\nu \beta_{4}x_{i}\frac{y_{n}}{N}-\left(\gamma +k\right)y_{sp}, \end{array}$$

where $N=y_{n}+y_{p}+y_{sp}$ and b, δ, ω, $\beta_{1},$ $\beta_{2},$ μ, $\beta_{3},$ $\beta_{4},$ γ, k, and ν are positive parameters.

The mathematical models governed by ordinary differential equations which are based on the compartmental methodology were introduced in the following 14 works from the retained list: [14,36,38,39,41,61,83,85,89,90,93,98,105,110]. The populations modeled in the different articles are diverse. More precisely, they are a plant population with susceptible, pollinated, and infected classes and a pollinator population divided into three classes, where they can carry neither pollen nor fungal spores, can carry pollen, or can carry spores [14]; healthy bees and impaired bees [36]; an uncapped brood, capped brood, hive bees, foragers, and food [38]; plants biomass with adult insects and adult insects with larvae [39]; a pollinator with pollen, pollinator without pollen, unpollinated flowering plants, and pollinated flowering plants [41]; the interactions of adult and non-adult pollinators [61]; four types of pollinators [83]; infected bumblebees, infected honeybees, and infected flowers [85]; the dynamics of viral genotypes [89]; contamined flowers, infected bees, and virus carrying [90]; a honeybee-–parasite interaction model with seasonality [93]; hive bees, unimpaired forager bees, and impaired forager bees [98]; the transmission dynamics of deformed wing virus in a honeybee colony infested with *Varroa* mites [105]; and three plants and two pollinators with juvenile, male, and female plant classes and insects [110].

### 3.2. Mathematical Models Based on Partial Differential Equations

The mathematical models based on partial differential equations were obtained by assuming the spatial movement of the pollinators. It is considered that the displacement of pollinators satisfies a diffusion law. Then, the partial differential equations are extensions of ordinary differential equation models. For instance, in the case of the interaction of two populations, the authors of [19] considered the ordinary differential equation for a pollinator–plant interaction system:

(16) $$\begin{array}{cc} \frac{da}{dt} & =a\left(K-a\right)+\frac{ap}{1+p}, \end{array}$$

(17) $$\begin{array}{cc} \frac{dp}{dt} & =-\frac{p}{2}+\frac{ap}{1+p}, \end{array}$$

where *K* is the carrying capacity for the pollinator population and *a* and *p* are the population densities of the pollinators and plants, respectively. Assuming that the pollinator population moves toward negative values of the gradient of the population density direction, the plants do not disperse, but their spatial distribution changes because of the interaction with the pollinator population. Then, the authors of [19] defined the new system extending the model in Equations (16) and (17) as follows:

(18) $$\begin{array}{cc} \frac{\partial a}{\partial t} & =D\Delta a+a\left(K-a\right)+\frac{ap}{1+p}, \end{array}$$

(19) $$\begin{array}{cc} \frac{\partial p}{\partial t} & =-\frac{p}{2}+\frac{ap}{1+p}, \end{array}$$

where the parameter D>0 is the diffusivity of the pollinator population and Δ is the Laplacian operator. Similarly, the authors of [19] assumed an ordinary differential model for pollinator–plant–herbivore interactions of the following type:

(20) $$\begin{array}{cc} \frac{da}{dt} & =ba\left(K-a\right)+\frac{g\left(h\right)k_{2}\mu \sigma ap}{1+\phi \sigma \mu^{2}p}, \end{array}$$

(21) $$\begin{array}{cc} \frac{dp}{dt} & =-\gamma p+\frac{g\left(z\right)k_{1}\mu \sigma ap}{1+\phi \sigma \mu^{2}p}-\frac{m_{1}ph}{c_{1}+p}, \end{array}$$

(22) $$\begin{array}{cc} \frac{dh}{dt} & =-\delta h+\frac{m_{2}ph}{c_{1}+p}, \end{array}$$

where *K* is the carry capacity for the pollinator population; a, *p*, and *h* are the population densities of pollinators, plants, and hervivores, respectively; *g* is a real function such that $g\in C^{1}\left[0,\infty \right),$ g(0)=1, in which $g^{\prime }\left(h\right)<0$ and g(h)>0 for all h>0, modeling the reduction rate of visits of pollinators to plants due to herbivore interaction; $k_{1}$ is the number of fertilized ova in each pollinator visit; σ is the probability of visits; ϕ is a measure of the speed of nectar extraction; and μ is the energetic recompense. The generalization of Equations (20)–(22) to a partial differential system is given by

(23) $$\begin{array}{cc} \frac{da}{dt} & =D_{1}\Delta a+ba\left(K-a\right)+\frac{g\left(h\right)k_{2}\mu \sigma ap}{1+\phi \sigma \mu^{2}p}, \end{array}$$

(24) $$\begin{array}{cc} \frac{dp}{dt} & =-\gamma p+\frac{g\left(z\right)k_{1}\mu \sigma ap}{1+\phi \sigma \mu^{2}p}-\frac{m_{1}ph}{c_{1}+p}, \end{array}$$

(25) $$\begin{array}{cc} \frac{dh}{dt} & =D_{2}\Delta a-\delta h+\frac{m_{2}ph}{c_{1}+p}, \end{array}$$

where the parameters $D_{1}>0$ and $D_{2}>0$ are the diffusivity of the pollinator and herbivore populations, respectively. Similar extensions of ordinary differential equations models were deduced via application of the compartmental methodology.

The mathematical models based on partial differential equations were considered in the following seven works: [19,26,27,31,32,50,111]. The modeled populations considered in the different articles are the following: the interaction of plant, pollinator, and herbivore populations [19,26]; harvester and scout populations Tyson [27]; multiple species of pollinators [31,32]; and plant and pollinator populations [50,111].

### 3.3. Network and Patch Mathematical Models

In the retained list, several works applied networks and patch concepts to model the dynamics of pollinator populations. In order to be precise, we consider [21], where the authors considered the interaction of a plant $p_{i}$ and animal $a_{j}$, obtaining the following system:

$$\begin{array}{cccc} \frac{dp_{i}}{dt} & =\sum_{j=1}^{n}\left(c_{ij}\frac{p_{i}a_{j}}{w_{j}}\right)\left(1-d-p_{i}\right)-e_{i}p_{i},\; & \; & 1\leq i\leq n,\\ \frac{da_{j}}{dt} & =c_{j}a_{j}\left(w_{j}-a_{j}\right)-e_{j}a_{j},\; & \; & 1\leq j\leq n, \end{array}$$

where $c_{ij}$ models the per capita colonization rate of a population of plants *i* when pollinated or dispersed by a pollinator *j*; $c_{j}$ is the per capita colonization rate of a pollinator *j*; $e_{i}$ and $e_{j}$ denote the per capita extinction rates for a plant *i* and animal *j*, respectively; *d* models the fraction of patches permanently lost through habitat destruction; and $w_{j}$ is the union of the patches occupied by *n* plant species interacting with the same *j* pollinator species.

In the retained list, we found that there were 20 works focused on the modeling of pollinator population dynamics using networks and patches [13,21,25,35,40,43,49,52,54,65,69,80,82,95,97,99,101,103,108,113]. In [13], the authors applied patch concepts to study the age-structured pollinator population model considering adult and non-adult pollinators [13]. Meanwhile, in the other works, the authors used networks and patch concepts [25,35,40,43,49,52,54,65,69,80,82,84,95,97,99,101,103,108,113].

### 3.4. Other Methodologies

Other articles that were difficult to include in the previous classification are the following 19 articles: [12,17,18,20,23,26,29,53,56,57,58,59,60,66,84,94,102,104,109]. These included a letter to the editor with an opinion on the mathematical model for mutualism on a patch [12], two review articles [26,102], a study on discrete models [20], a work focused on the study of virulence [23], a study on microscopic populations by considering five types of cells [17], a study on the fractional order mathematical mould for plant–pollinator–nectar interactions [84], a study on the modelization of pollen transport [58], studies on the concept of delaying ordinary differential equations to model a plant-pollinator system [59,94], a study on adult and juvenile pollinators [104], a study on the idealization of bumble bees [56], studies on the application of stochastic differential equations [18,109], a study on the analysis of flowering [29], studies on the modellization by hybrid ordinary differential equations and partial differential equations [53,60], a study on a particular form of ordinary differential system for modeling genotypes [57], and an empirical study which developed data fitting for ordinary differential models [66].

### 3.5. A Summary of the Topics Studied in the Retained List

The analysis and main results of the articles of the retained list focused on the following seven topics:

(1) *Positive bounded solutions*: The variables of the mathematical models are the population or the density of the population. Then, the first question of the consistence of the mathematical model with the biological system is for analyzing if the mathematical model’s solutions are positive and bounded. In this sense, the following works [15,19,20,26,28,30,41,50,54,59,65,66,71,75,78,93,102] have explicit results proving that the dynamics of the mathematical systems have positive bounded solutions.
(2) *Equilibrium and stability analysis*: In the mathematical analysis of dynamical systems, the study of linearization and asymptotic behavior is strongly related to the analysis of stability analysis. In particular, mathematical models are an important tool for characterizing the large time behavior of the system and answering other important questions, like the prevalence or extinction a species of pollinator. The works focused on the development of equilibrium and stability analysis are the following [10,11,12,14,15,19,20,22,26,33,34,36,39,43,44,45,46,47,48,54,55,57,60,61,63,67,68,70,71,73,74,75,76,78,79,81,84,86,90,91,92,93,95,96,98,100,102,103,104,107,111,113,114].
(3) *Bifurcation*: One topic related to equilibrium and stability analysis is bifurcation analysis. Indeed, the analysis of bifurcation was introduced in [16,30,53,67,74,94,103,106,107,114].
(4) *Mutualistic interactions*: In the case of mathematical models based on networks and patch concepts, there are several topics which have been researched, including coexistence [13,16,18,21,24,25,28,30,33,35,37,39,40,43,44,45,46,51,52,53,54,62,64,65,68,69,70,71,72,80,81,82,92,96,97,99,100,101,103,108], dissipation [28,33,34,48,74,78,94], and eco-evolution [59,67,68,94].
(5) *Periodicity of the solution*: An interesting question for pollinators strongly related with seasonality is what the periodicity behavior of the populations of the different variables involved in pollination models is. Indeed, the following topics have been researched: periodic orbits [30,47,65,93], non-periodic orbits [28,45,48,65,74], and oscillation [30,53,63].
(6) *Numerical solutions and comparison with empirical data*: The mathematical models are strongly nonlinear, and the analytical solution cannot be construed. Consequently, numerical solutions of the mathematical models are introduced in order to simulate and calibrate the mathematical models. In the retained list, the authors of [19,25,26,31,32,35,36,37,42,53,56,57,58,60,61,66,67,68,69,75,78,84,85,87,88,89,90,92,94,96,98,99,100,101,103,104,105,106,108,110,111,113,115,116] developed numerical simulations.
(7) *Mathematical control*: Optimal control of the pollination systems via introducing appropriate control variables was conducted in [88,97].

## 4. Biological and Applied Problem Typologies in the Retained Literature

The retained articles address a spectrum of biological and applied problems through mathematical modeling. These can be categorized into seven thematic domains, each reflecting distinct modeling priorities, parametrization strategies, and implications for ecological policy and management:

(1) *Biological consistency and population viability*: In this group, we consider the works addressing biological realism in population dynamics and focus on the research of positive bounded solutions. Models in this category ensure that population variables remain biologically meaningful, i.e., non-negative and bounded over time. This foundational consistency is critical for validating ecological interpretations and avoiding spurious predictions. Parametrization typically involves biologically constrained initial conditions and growth functions (e.g., logistic or saturating terms). These models support policy decisions related to conservation thresholds and population viability. Representative works include [15,19,20,26,28,30,41,50,54,59,65,66,71,75,78,93,102].
(2) *Long-term dynamics and species persistence:* In this group, the problems to study are prevalence, extinction, and asymptotic behavior. These studies examine the conditions under which pollinator populations persist or collapse, often through linearization techniques and Lyapunov-based stability criteria. Parametrization emphasizes sensitivity to reproductive rates, mortality, and interaction coefficients. The results inform long-term sustainability planning and resilience forecasting. Representative works include [10,11,12,14,15,19,20,22,26,33,34,36,39,43,44,45,46,47,48,54,55,57,60,61,63,67,68,70,71,73,74,75,76,78,79,81,84,86,90,91,92,93,95,96,98,100,102,103,104,107,111,113,114].
(3) *Regime shifts and critical transitions*: In this group of works, the authors focus on the bifurcation analysis and address the study of threshold phenomena and qualitative change. Bifurcation studies identify parameter regimes where small changes induce qualitative shifts in system behavior, such as transitions from coexistence to extinction. These models often employ continuation methods and bifurcation diagrams to explore critical thresholds, with implications for adaptive management and early warning indicators. Representative works include [16,30,53,67,74,94,103,106,107,114].
(4) *Mutualism and network structure*: In this group, the focus is mutualistic interactions and the study of phenomena like coexistence, dissipation, and eco-evolutionary dynamics. These models incorporate spatial structure, network topology, and evolutionary feedback to explore how mutualistic systems maintain biodiversity. Parametrization includes patch-based connectivity, trait evolution, and interaction matrices. The findings support the design of pollinator corridors, agroecological zoning, and biodiversity incentives. Representative works include [13,16,18,21,24,25,28,30,33,35,37,39,40,43,44,45,46,51,52,53,54,62,64,65,68,69,70,71,72,80,81,82,92,96,97,99,100,101,103,108], as well as dissipation-focused studies [28,33,34,48,74,78,94], and eco-evolutionary dynamics studies [59,67,68,94].
(5) *Seasonal and oscillatory behavior*: The addressed problem is the temporal variability and seasonality, along with the study of periodicity and oscillations in model solutions. Models in this group address how seasonal forcing and intrinsic dynamics lead to periodic or chaotic population fluctuations. Parametrization incorporates time-dependent coefficients and delay terms. These insights guide seasonal pollination services, crop planning, and phenological synchronization. Representative works include [28,30,45,47,48,53,63,65,74,93].
(6) *Simulation and empirical calibration:* In this group, we consider works focused on numerical solutions and data comparison and developed for model validation and empirical integration. Due to nonlinear complexity, many models rely on numerical simulations to explore parameter spaces and fit empirical data. Parametrization strategies include optimization techniques, sensitivity analysis, and empirical calibration. These models enhance the credibility of model-based recommendations and support data-driven decision making. Representative works include [19,25,26,31,32,35,36,37,42,53,56,57,58,60,61,66,67,68,69,75,78,84,85,87,88,89,90,92,94,96,98,99,100,101,103,104,105,106,108,110,111,113,115,116].
(7) *Intervention and optimization*: There are some works on solving the problem of applied control and resource allocation, which are focused on mathematical control. These studies introduce control variables—such as habitat enhancement or pesticide reduction—to optimize ecological outcomes. Parametrization uses Pontryagin’s maximum principle or dynamic programming to derive optimal strategies. The results directly inform cost-effective conservation and adaptive management protocols (see [88,97]).

## 5. Other Aspects of the Literature Review

### 5.1. Research Gaps and Future Directions for Control, Stochastic Modeling, and Network-Based PDEs

Despite the breadth of topics addressed in the retained literature, three modeling domains remain notably underdeveloped: (1) optimal control under uncertainty, (2) stochastic ecological modeling, and (3) network- or patch-based partial differential equations (PDEs) for spatially structured systems. These gaps are particularly relevant given the increasing complexity of ecological systems and the need for robust, data-informed decision making:

(1) *Optimal Control under Uncertainty:* While mathematical control was explored in [88,97], current models rely on deterministic frameworks and assume full observability of system states and parameters. These assumptions limit applicability in real-world settings, where ecological responses to interventions (e.g., pesticide reduction or habitat restoration) are uncertain and data are sparse. Neither study incorporated stochastic perturbations or feedback mechanisms, nor did they address parameter uncertainty or adaptive control strategies. This restricts the robustness and generalizability of the proposed solutions.
(2) *Stochastic Modeling*: Across the retained list, stochastic formulations are conspicuously absent. Although several studies addressed oscillatory behavior and bifurcation phenomena (e.g., [30,53,63]), they did so within deterministic systems. The lack of stochastic differential equations or probabilistic transitions limits the capacity to model demographic noise, environmental variability, and uncertainty propagation, especially in fragmented landscapes or under climate stress. This gap is critical given the increasing emphasis on resilience and risk-aware ecological planning.
(3) *Network-Based PDEs and Patch Dynamics*: Numerous studies incorporated network or patch structures in mutualistic systems (e.g., [13,21,28,33,40,65,71,72,96,100]), yet most relied on discrete or compartmental models. Continuous-space PDEs on networks or graph-based domains are rare, and when present, they often lack empirical calibration or realistic topologies. For example, the authors of [28,48,74] explored dissipation and spatial dynamics but did not integrate high-resolution landscape data or adaptive dispersal mechanisms. This limits the ecological realism and policy relevance of spatial predictions.

### 5.2. Limitations of Current Findings

Across these domains, a recurring limitation is the scarcity of longitudinal, high-resolution data for model calibration and validation. Many studies rely on synthetic simulations (e.g., [67,68,69,75,78]) or static parameter estimates, which constrain ecological realism and hinder generalization across systems. Furthermore, sensitivity analysis and uncertainty quantification are rarely formalized, reducing the interpretability and robustness of model outcomes. Additionally, we report at least three limitations of this research: a checklist for data extraction was not constructed, only two databases were considered, and the analysis of data was developed without using advanced methodologies.

### 5.3. Methodological Pathways

To address these gaps, future research should pursue hybrid frameworks that integrate stochasticity into control models (e.g., stochastic optimal control or robust model predictive control), and embed network-aware partial differential equations within empirically grounded landscapes. Promising techniques include the following:

(1) Graph Laplacians and metapopulation partial differential equations for dispersal modeling;
(2) Bayesian inference and ensemble simulations for uncertainty quantification;
(3) Data assimilation methods for real-time calibration.

### 5.4. Potential Data Sources

Empirical grounding can be strengthened using the following:

(1) Remote sensing data for habitat fragmentation and land use change;
(2) Citizen science platforms (e.g., iNaturalist or eBird) for species occurrence;
(3) Long-term ecological monitoring networks (e.g., Global Biodiversity Information Facility—GBIF, or Long Term Ecological Research—LTER) for population dynamics.

### 5.5. Roadmap for Future Work

A strategic agenda should include the following:

(1) Development of modular, interoperable modeling platforms that integrate control, stochasticity, and spatial structure;
(2) Co-design of models with stakeholders to ensure contextual relevance and usability;
(3) Formal incorporation of sensitivity analysis and uncertainty quantification;
(4) Establishment of typological benchmarks to compare model performance across ecological and socio-political scenarios.

Such efforts will enhance both theoretical rigor and translational impact, positioning mathematical ecology as a key contributor to adaptive management and evidence-based policy design.

### 5.6. Implications of the Retained Modeling Topics for Agricultural Planning, Habitat Management, and Pesticide Regulation

The seven modeling themes identified in Section 3.5 provide a rigorous mathematical foundation for informing real-world decision making in agroecological systems. Their relevance extends to agricultural planning, habitat conservation, and the formulation of pesticide policies. Below, we detail the practical implications of each topic:

(1) *Positive Bounded Solutions:* Ensuring that model solutions remain positive and bounded is essential for biological realism, particularly when variables represent population densities. This property supports the development of ecologically valid simulations that can guide agricultural interventions and pesticide thresholds, preventing unintended population collapses.
(2) *Equilibrium and Stability Analysis*: Stability analysis enables the characterization of long-term system behavior, including species persistence or extinction. In agricultural contexts, it informs crop-pollinator compatibility and resilience, while in habitat management, it supports the design of restoration strategies and ecological corridors.
(3) *Bifurcation Analysis*: Bifurcation theory reveals how small parameter changes can induce qualitative shifts in system dynamics. This is critical for anticipating nonlinear responses to environmental stressors, such as pesticide application or habitat fragmentation, and for designing adaptive management strategies that avoid tipping points.
(4) *Mutualistic Interactions*: Modeling mutualistic networks elucidates mechanisms of coexistence, dissipation, and eco-evolutionary dynamics. These insights inform the diversification of cropping systems, the conservation of keystone mutualists, and the regulation of agrochemicals that may disrupt ecological interactions.
(5) *Periodicity of Solutions*: Seasonal and periodic behaviors in pollinator populations are central to synchronizing agricultural calendars with ecological cycles. Understanding periodicity aids in optimizing planting schedules, flowering periods, and pesticide applications to align with pollinator activity.
(6) *Numerical Simulations and Empirical Validation*: Given the nonlinear nature of most models, numerical simulations are indispensable for calibration and scenario testing. These simulations support evidence-based agricultural planning and policy evaluation, enabling cost-benefit analyses of proposed interventions.
(7) *Mathematical Control*: Optimal control frameworks allow for the strategic modulation of system variables to achieve desired ecological or economic outcomes. In agriculture, this translates to resource-efficient practices that sustain pollinator populations, while in regulatory contexts, it supports dynamic policy design responsive to ecological feedback.

Collectively, these modeling approaches bridge theoretical ecology with applied decision making, offering quantitative tools for sustainable land use, biodiversity conservation, and environmental governance.

### 5.7. A Particular Comparative Analysis

In this subsection we develop a comparative analysis between [74] and the Thematic Synthesis in Section 3.5.

The authors of [74] offered a focused and technically rigorous contribution to the mathematical modeling of ecological systems, particularly in the context of bifurcation analysis, dissipative dynamics, and non-periodic oscillatory behavior. Chen’s work employed nonlinear differential equations to explore critical transitions and qualitative shifts in population dynamics, with emphasis on parameter sensitivity and system resilience. The model demonstrates how small perturbations can lead to significant changes in ecological outcomes, contributing to the literature on regime shifts and early warning indicators.

However, when compared with the broader synthesis presented in Section 3.5, the scope of [74] appears more specialized and thematically constrained. The retained literature encompassed seven interrelated modeling domains, ranging from positive boundedness and stability analysis to mutualistic networks, seasonality, empirical calibration, and optimal control. This thematic architecture enables a more comprehensive understanding of pollinator dynamics and ecological decision making.

Notably, the synthesis in Section 3.5 integrates multiple methodological layers:

- It links biological realism (e.g., positive bounded solutions [15,19]) with long-term system behavior (e.g., stability analysis [10,44]).
- It incorporates spatial and network structure in mutualistic interactions [21,33], extending beyond the local dynamics emphasized in [74].
- It addresses empirical calibration and numerical simulation [68,75], which are not central in Chen’s formulation.
- It introduces optimal control frameworks [88,97], offering policy-relevant strategies absent in [74].

In this light, the novelty of Section 3.5 lies in its integrative typology, which not only categorizes the retained studies but also reveals methodological synergies and thematic gaps. While [74] contributes valuable insights into bifurcation and dissipation phenomena, the broader synthesis provides a multidimensional roadmap for future research—bridging theoretical modeling with empirical validation and policy design.

This comparative perspective underscores the importance of typological frameworks in advancing ecological modeling, enabling researchers to situate individual studies within a structured landscape of methodological and applied relevance.

## 6. Conclusions

In this paper, we applied a systematic bibliographic review. Our research methodology adeptly allowed us to identify and analyze a substantial body of research on the mathematical modeling of pollinators. We retrieved and reviewed 107 works published between 1981 and 2025, leveraging databases such as the Web of Science and Mathscinet. We examined the mathematical theory and the topics analyzed. Our findings reveal a significant increase in research dedicated to the introduction or improvement of mathematical modeling to study the dynamics of pollinators. The landscape of mathematical modeling of pollinators has covered the standard topics of dynamical systems, like equilibrium and stability analysis. However, in recent years, the field has shifted dramatically, moving away to include some new topics like the fractional-order or diffusion models. In particular, modeling by using networks has promise in the future development of research. Moreover, given that the mathematical models arise from different mathematical approaches, it is essential to use an interdisciplinary approach for constructing complex models that more closely resemble pollination phenomena.

Research on the mathematical modeling of pollinators is an active area. However, there is still much to be developed in the context of addressing the challenges of pollination dynamics. We identified four issues that require further detailed exploration. First, there is the study of mathematical control theory about biological control. We identified only two works related to control theory (see [88,97]). Therefore, constructing mathematical models that incorporate the principles of optimal control is necessary. Second, the development of stochastic models is an area that needs strong attention from researchers. Third, the inclusion of network and patch concepts with mathematical models based on partial differential equations is a topic that requires attention and future development. Fourth, we reduced the present analysis to the word pollinators and two databases. Clearly, some representative works researching mathematical models for pollinators were excluded. We plan to expand our search to other specific pollinators and to other bibliographic databases, including Scopus, PubMed, BIOSIS Previews, and AGRICOLA.

## Figures and Tables

**Table 1:** The nine journals in the first four positions, considering the number of articles published.

Rank	Journal	Record Count	% of 106
1°	*Journal of Theoretical Biology*	12	11.32
2°	*Bulletin of Mathematical Biology*	8	7.55
3°	*Ecology Letters*	4	3.7
	*International Journal of Biomathematics*	4	3.77
	*Theoretical Ecology*	4	3.77
4°	*Journal of Mathematical Biology*	3	2.83
	*Oikos*	3	2.83
	*Plos One*	3	2.83
	*Theoretical Population Biology*	3	2.83

**Table 2:** The top 10 journals based on the H index, SJR index, and quartile. The information was obtained from Scimago https://www.scimagojr.com/ (accessed on 20 April 2025). Refer to Table A2 for the complete list of journals.

Rank	Journal	H Index	SJR 2024	Quartile SJR	Subject Area and Category
1°	*Proceedings of the National Academy of Sciences of the United States of America*	896	10.8	Q1	Multidisciplinary Sciences
2°	*Plos One*	467	3.3	Q1	Multidisciplinary Sciences
3°	*Scientific Reports*	347	4.3	Q1	Multidisciplinary Sciences
4°	*Ecology*	343	5.5	Q1	Ecology
5°	*Ecology Letters*	330	9.8	Q1	Ecology
6°	*Plos Pathogens*	260	5.5	Q1	Microbiology, Parasitology
7°	*American Naturalist*	236	3.3	Q2	Ecology
8°	*Evolution*	227	3.0	Q2	Ecology/Evolutionary Biology
9°	*Journal of Ecology*	219	6.1	Q1	Ecology, Plant Sciences
10°	*Journal of Applied Ecology*	216	6.2	Q1	Biodiversity Conservation, Ecology

**Table 3:** The top four authors with the highest number of articles in the selected list.

Author	Institution	Number of Articles
Yuanshi Wang	Sun Yat-sen University, P. R. China	16
Hong Wu	Sun Yat-sen University, P. R. China	7
Faustino Sánchez-Garduño	Universidad Nacional Autónoma de México, Mexico	6
Fernanda S. Valdovinos	University of California Davis, USA	4

**Table 4:** Summary of mathematical model topics and phenomena studied in the retained list of papers. Here, RR stands for retained reference.

RR	Model Type	Assumptions	Parameterization	Validation	Key Findings
[10]	Ordinary differential equations of Lotka–Volterra type	Two-species, mutualistic interaction, closed system, constant environment	Interaction coefficients derived from the ecological literature; intrinsic growth rates assumed to be constant	Analytical exploration of equilibrium points and stability, qualitative phase plane analysis	Demonstrated conditions for mutualistic coexistence and thresholds for collapse due to partner dependency
[11]	Ordinary differential Lotka–Volterra equations with ecological feedback	Mutualistic interaction of pollinators and plants, continuous population dynamics, homogeneous environment	Growth rates and interaction, coefficients estimated from empirical observations and the ecological literature	Local stability analysis, numerical simulations exploring population trajectories under varying scenarios	Identification of stable equilibria, limit cycles, and bifurcation points explaining persistence or collapse of mutualistic systems
[12]	Discrete patch-based mutualism model	Species occupy spatially distinct patches; mutualistic benefit depends on local density; extinction and colonization are patch-specific	Parameters included colonization rate, extinction probability, and mutualistic benefit per patch; values treated generically for theoretical exploration	Analytical derivation of equilibrium conditions; stability assessed via local perturbation analysis	Mutualism enhances patch occupancy and persistence; spatial structure stabilizes interactions; coexistence possible without obligate dependence
[13]	Patch-based metapopulation model	Species occupy discrete habitat patches; extinction risk decreases with population size; colonization depends on local density	Extinction and colonization rates modeled as functions of population size; parameters derived from ecological theory	Analytical derivation of equilibrium distributions; stability analysis of patch ensemble	Demonstrated that spatial structure stabilizes mutualistic systems and intra-patch dynamics critically influence metapopulation persistence
[14]	Compartmental disease model with pollination-mediated transmission	Vector-borne fungal disease spreads via pollinators; host recruitment and pollinator visitation drive transmission	Transmission potential and recruitment rates estimated from empirical data; no explicit density dependence	Analytical threshold conditions for disease invasion; bifurcation analysis of host–pathogen dynamics	Identified conditions for disease-induced host extinction; showed that high disease incidence suppresses pollination and drives collapse
[15]	Three-species ordinary differential equations model (herbivore, plant, and pollinator)	Non-obligate mutualism; herbivory reduces pollinator visitation; population dynamics are continuous and deterministic	Functional responses and interaction coefficients derived from the ecological literature; visitation rates modeled explicitly	Stability analysis of equilibria; numerical simulations of population trajectories	Showed that herbivory can indirectly promote pollinator persistence; identified conditions for coexistence and oscillatory dynamics
[16]	Adaptive dynamics with piecewise smooth bifurcation structure	Evolution of mutualistic traits constrained by physiological costs; trait space bounded; bifurcations occur at viability borders	Trait-dependent fitness functions and bifurcation parameters derived from evolutionary stability conditions	Analytical and numerical bifurcation analysis; detection of border collision bifurcations	Revealed abrupt evolutionary transitions in mutualism; identified critical thresholds for trait viability and coexistence
[17]	Cellular automata with pair approximation	Pollination and reproduction modeled as separate processes; spatial clumping affects contact rates; Allee effects are context-dependent	Local and global interaction rules encoded in probabilistic automata; pair approximation used to derive ODE caricatures	Comparison of pair approximation predictions with full simulation outcomes	Demonstrated that spatial structure can eliminate Allee effects; local pollination and colonization enhance reproductive success in small populations
[18]	Individual-based spatial model with evolutionary dynamics	Obligate mutualism between plant and pollinator; parasitoid antagonist and dispersal evolves under selection; homogeneous landscape	Dispersal kernels, visitation rates, and mortality probabilities, parameters derived from ecological data and theoretical distributions	Simulation-based pattern formation; comparison with non-spatial ordinary differential equation model; sensitivity to obligacy constraints	Identified evolutionarily stable dispersal distances; showed that obligacy promotes spatial patterning and coexistence and weak obligacy leads to collapse of spatial structure
[19]	Reaction, diffusion, advection partial differential equation system	Three-species system: plant, pollinator, herbivore; nonlinear interactions; spatial movement and local reactions; continuous time and space	Diffusion and advection coefficients; interaction terms inspired by physical analogies; parameters selected for dynamical richness	Analytical treatment of reduced subsystems; numerical simulations of full spatiotemporal model	Demonstrated emergence of spatial heterogeneity and traveling waves; showed that mutualism and antagonism interact to produce complex spatial patterns
[20]	Three-species ordinary differential equation model with asymmetric mutualism	One plant species with two pollinators differing in efficiency and genetic impact; mutualism and competition coexist; inbreeding affects seed viability	Growth rates, nectar consumption rates, and pollination success calibrated from biological traits; inbreeding modeled as a dynamic feedback	Stability analysis and bifurcation exploration; numerical simulations under varying asymmetry and inbreeding levels	Found that pollinator asymmetry enhances system stability; high inbreeding paradoxically increases population persistence; oscillatory regimes linked to trait divergence
[21]	Stochastic metacommunity network model	Pollination network includes native and alien plants; mutualistic interactions structured by network topology; species abundances evolve over time	Degree distribution, nestedness, and modularity used to define network structure; demographic parameters estimated from field data	Simulations of network dynamics under species removal; comparison of full vs. native-only networks	Removal of alien plants destabilizes network structure and reduces species persistence; network topology strongly influences long-term dynamics and resilience
[22]	Multi-scale ordinary differential equation model with individual-to-population extrapolation	Pollinator foraging occurs at multiple temporal scales; mutualism affects reproduction and survival; population-level dynamics derived from individual-level interactions	Parameters derived from empirical foraging behavior and floral handling times; interaction terms approximated via Beddington–DeAngelis functional response	Analytical derivation of equilibrium conditions; numerical simulations of long-term dynamics	Demonstrated bi-stability and threshold effects in plant-pollinator systems; highlighted the role of individual-level behavior in shaping population-level outcomes
[23]	Conceptual coevolutionary framework with tolerance–virulence trade-offs	Host fitness is reduced by parasite virulence; tolerance evolves independent of resistance; coevolution affects both host and parasite traits	Virulence modeled as a function of parasite density and per-parasite damage; host fitness expressed as a linear function of tolerance and infection burden	Theoretical synthesis of existing models; conceptual validation through comparative analysis of empirical cases	Proposed that tolerance can drive parasite counter-adaptation; emphasized the need for integrated models of host–parasite coevolution, including mutualistic analogs
[24]	Game theoretic model of aggression in multi-partner mutualism	Plants interact simultaneously with ants and pollinators; ant aggression affects pollinator survival; fitness interests are misaligned across partners	Aggression modeled as a continuous trait; payoff matrices constructed for plant, ant, and pollinator strategies; parameters derived from ecological scenarios	Analytical derivation of evolutionary stable strategies; threshold analysis of aggression levels	Identified conditions under which pollinators are excluded due to ant aggression; facultative mutualisms more vulnerable than obligate ones; proposed empirical tests for aggression thresholds
[25]	Network-based dynamical model with topological asymmetry	Mutualistic networks are asymmetric; specialists interact with generalists, habitat destruction removes nodes and links, and extinction cascades depend on network structure	Network topology defined by degree distribution and nestedness; demographic parameters estimated from empirical pollination networks	Simulations of network collapse under progressive habitat loss; entropy metrics used to assess differential susceptibility	Found that disassortative networks buffer specialist species against extinction; asymmetry promotes resilience under habitat fragmentation; validated predictions with real-world network data
[26]	Reaction, diffusion, and advection system	Three-species system (plant, pollinator, herbivore); nonlinear interactions; spatial movement via diffusion and advection; Holling type II functional response	Diffusion and advection coefficients selected for dynamical richness; interaction terms derived from ecological analogies	Analytical proof of existence, positivity, and boundedness; numerical simulations under Dirichlet and Neumann boundary conditions	Showed emergence of spatial patterns and traveling waves; mutualism and antagonism jointly shape spatial heterogeneity and coexistence
[27]	Hybrid dispersal model with intensive and extensive search modes	Foraging population divided into two behavioral states—intensive (local) and extensive (long range)—with movement modeled via diffusion and advection, respectively	Search mode parameters calibrated from empirical movement data; dispersal kernels constructed from behavioral observations	Comparison with traditional diffusion models; simulation-based fit to spatial distribution data	Demonstrated superior predictive power of hybrid model; emphasized behavioral heterogeneity as key to dispersal dynamics and ecological forecasting
[28]	Three-species ordinary differential equation model with unidirectional consumer–resource interaction	Plant provides nectar to both pollinator and nectar robber; pollinator offers mutualistic service, but robber is exploitative; interaction asymmetry drives coexistence	Functional responses tailored to mutualist and exploiter; parameters derived from empirical studies and theoretical ecology	Stability and persistence analysis; numerical simulations of invasion scenarios and equilibrium dynamics	Identified conditions for stable coexistence despite exploitation; showed that pollinator’s dual role (beneficial and costly) mediates system resilience
[29]	Eco-evolutionary model of mutualism under climate change	Plant and pollinator phenologies evolve in response to climate shifts; mutualism persistence depends on synchrony and community composition	Evolutionary rates and phenological traits modeled explicitly; alternative partner availability incorporated as dynamic variables	Simulation of evolutionary trajectories under varying climate scenarios; sensitivity analysis of Allee thresholds	Found that mutualism robustness depends on partner diversity and temporal overlap; identified thresholds beyond which climate-induced asynchrony leads to collapse
[30]	Three-species ordinary differential equation model with mutualism and exploitation	Plants interact with pollinators (mutualism) and nectar robbers (parasitism); functional responses differ across interactions and population-level dynamics	Beddington–DeAngelis and Holling type II responses; parameters derived from the ecological literature and theoretical constraints	Analytical conditions for persistence and extinction; bifurcation analysis of coexistence regimes	Identified mechanisms for stable coexistence in presence of robbers; showed that mutualism can persist despite exploitation under specific parameter thresholds
[31]	Cross-diffusion partial differential equation model with empirical calibration	Honey bees and solitary bees forage in almond orchards; movement influenced by environmental favorableness and interspecific interactions	Shigesada–Kawasaki–Teramoto framework; parameters calibrated from field data and canopy structure; spectral Galerkin method used for numerical approximation	Numerical simulations of spatial redistribution; empirical validation via bee visitation data and pollen movement patterns	Demonstrated that cross-diffusion leads to increased inter-tree movement and enhanced cross-pollination; model supports use of diverse pollinator assemblages to improve yield
[32]	Two-species SKT model with habitat choice and productivity feedback	Pollinators choose habitats based on floral density and competition; movement modeled via cross-diffusion; productivity linked to pollen transfer efficiency	SKT model applied to almond trees; favorableness gradients derived from floral distribution; interspecific effects modeled explicitly	Spectral Galerkin simulations; sensitivity analysis of pollinator redistribution and productivity outcomes	Found that interspecific competition drives honey bees into less favorable zones, increasing cross-pollination; spatial heterogeneity enhances productivity in mixed-variety orchards
[33]	Three-species ordinary differential equation model with cheater invasion	Plants classified as nectar secretors or non-secretors; pollinators interact mutualistically with secretors and parasitically with cheaters; fitness depends on nectar availability	Beddington–DeAngelis functional responses for both interactions; efficiency thresholds defined for cheater invasion	Global stability analysis; threshold conditions for persistence and extinction; numerical simulations of invasion dynamics	Showed that nectarless flowers can invade and persist under specific efficiency conditions; identified scenarios where cheaters drive mutualists to extinction, leading to system collapse
[34]	Ordinary differential equation model with mutualism and parasitism	Plants and pollinators form a mutualistic pair; nectar robbers exploit plants without providing pollination; pollinators and robbers share a limiting resource without direct interference	Functional responses include Beddington–DeAngelis and Holling type II; parameters varied to explore invasion and persistence scenarios	Analytical derivation of equilibria and stability conditions; numerical simulations of coexistence, extinction, and invasion dynamics	Mutualism can persist despite robber invasion; coexistence possible under intermediate robber efficiency and favorable initial conditions; pollinators not necessarily driven to extinction by robbers
[35]	Consumer–resource ordinary differential equation model with adaptive foraging	Pollinators adjust foraging efforts based on floral reward availability; network structure influences interaction strength; mutualism is dynamic and plastic	Empirical pollination networks used to calibrate interaction matrices; adaptive foraging implemented via optimization routines	Simulations of network dynamics under species loss; comparison of static vs. adaptive foraging scenarios	Adaptive foraging enhances biodiversity and network robustness and reduces secondary extinctions by promoting niche partitioning among pollinators and plants
[36]	Compartmental model of colony impairment under sublethal stress	Sublethal pesticide exposure impairs individual bees without causing mortality; colony function depends on cumulative impairment; positive density dependence drives collapse	Parameters derived from empirical exposure experiments; impairment modeled as transition between healthy and dysfunctional states	Model fitted to experimental data from bumblebee colonies; comparison with non-impairment models	Sublethal stress leads to colony failure via feedback loops; impairment thresholds generate Allee effects and bistable dynamics; model explains enigmatic patterns of collapse
[37]	Three-species consumer–resource model with indirect interactions	Plant provides nectar to mutualist (pollinator) and parasite (robber); indirect interactions emerge via shared resources; feedback loops modulate coexistence	Functional responses calibrated from empirical nectar consumption rates; interaction strengths derived from ecological theory	Analytical derivation of equilibrium and limit cycles; simulations of indirect interaction strength and persistence	Mutualism stabilizes food web module despite parasitism; (+,–) indirect interactions promote coexistence; density-dependent feedback enhances resilience
[38]	Compartmental population model of honey bee colony dynamics	Colony composed of brood, hive bees, and foragers; food availability regulates development and foraging onset; mortality and recruitment are stage-specific	Parameters derived from empirical data on bee life stages and foraging behavior; food dynamics modeled via differential equations	Simulations of colony trajectories under varying food and mortality rates; equilibrium and threshold analysis	Identified critical thresholds for forager mortality beyond which colonies collapse; food availability buffers colony resilience; model supports predictive management of hive health
[39]	Stage-structured ordinary differential equation model with facultative obligate mutualism	Plant is facultative; pollinator is obligate with life stage structure; external stressors affect larval development and adult survival	Life stage transitions modeled explicitly; demographic rates derived from insect biology and the ecological literature	Analytical stability analysis; bifurcation exploration under varying demographic dominance	Identified hysteresis and collapse thresholds; pollination service vulnerable to shifts in pollinator structure; recovery requires large demographic compensation
[40]	Three-dimensional autonomous ordinary differential equation system with cooperative and competitive interactions	Two plant species compete, one pollinator interacts cooperatively with both, and biodiversity emerges from interaction topology	Growth rates, competition coefficients, and mutualistic terms defined via mean-field approximations; handling time neglected	Analytical and numerical exploration of attractors and limit cycles; comparison with reduced competitive system	Demonstrated that cooperative species enhance biodiversity even when driven to extinction; proposed structural vulnerability of key mutualists
[41]	Hybrid dynamical model with seasonal and intra-seasonal phenology	Plant and pollinator phenologies respond differently to climate change; demographic outcomes depend on synchrony and lifespan	Zonneveld-type non-autonomous ordinary differential equations for within-season dynamics and discrete-time equations for seasonal transitions	Zonneveld-type non-autonomous ordinary differential equations for within-season dynamics and discrete-time equations for seasonal transitions	Found that short-lived species are highly sensitive to mismatching; hybrid models capture demographic consequences of climate-driven phenological shifts
[42]	Reaction, diffusion, and advection system with nonlinear functional responses	Three interacting populations: plant, pollinator, and herbivore; spatial movement via diffusion and advection; non-monotonic response functions	Parameters selected for dynamical richness; functional responses derived from ecological analogies and prior models	Analytical reduction to autonomous ordinary differential equation system; numerical simulations of full spatiotemporal model	Herbivore stabilizes mutualistic dynamics; coexistence enhanced by spatial heterogeneity; limit cycles and attractors depend on interaction strength
[43]	Lotka–Volterra cooperative system with delay	One hyper-connected mutualistic species interacts with multiple peripheral species; delays represent interaction lags; no inter-peripheral interactions	Birth rates and interaction coefficients assumed to be positive; delays modeled via distributed kernels	Lyapunov functionals used to prove global stability; analytical derivation of coexistence conditions	Demonstrated global asymptotic stability of coexistence equilibrium; extended classical Lotka-–Volterra framework to nested mutualistic networks
[44]	Three-species ordinary differential equation model with dual mutualism and interference	Plant interacts mutually with both pollinator and ant; ant interferes with pollinator access; functional responses differ across interactions	Beddington–DeAngelis for mutualisms and Holling type II for interference; parameters derived from the ecological literature	Stability analysis and threshold exploration; numerical simulations of coexistence and extinction regimes	Identified threshold in ant aggressiveness; coexistence possible under weak interference; strong interference leads to pollinator extinction and mutualism collapse
[45]	Extended Beddington–DeAngelis model with asymmetric mutualism	Pollinators and ants interact with plants; ants interfere with pollinators but depend on mutualism for survival, with feedback loops included	Functional responses extended to capture interference; parameters derived from empirical studies and theoretical constraints	Global dynamics analyzed via persistence theory; bifurcation analysis of extinction thresholds	Showed that mutualism can persist under moderate interference; strong ant dependence on pollination stabilizes coexistence; extinction cascades occur under high interference
[46]	Three-species dynamical system with extended functional responses	Plant–pollinator and plant-–ant interactions are mutualistic; ant interference modeled explicitly; system includes indirect effects	Extended Beddington-–DeAngelis responses; interaction strengths calibrated from ecological theory and prior models	Analytical derivation of boundary equilibria; numerical simulations of persistence and extinction dynamics	Defined threshold for ant interference; weak interference promotes mutualism synergy; strong interference destabilizes entire system, including ant population
[47]	Ordinary differential equation model with pollinator learning and expertise differentiation	Pollinators are divided into novice and expert classes; learning improves pollination efficiency; plant growth benefits from expert visitation	Logistic growth for plants; mutualistic benefit modeled via saturating functional response; learning encoded via efficacy parameter σ	Analytical derivation of equilibrium and stability; numerical simulations of coexistence dynamics	Showed stable coexistence of plants, novices, and experts; learning enhances mutualistic benefit and system resilience; expertise evolution supports pollination service
[48]	Three-species ordinary differential equation model with nectar robbing	Pollinators provide mutualistic service; nectar robbers consume floral resources without pollination; robbers compete indirectly with pollinators	Beddington–DeAngelis functional responses for both mutualism and exploitation; parameters derived from ecological theory	Stability and persistence analysis; bifurcation exploration of coexistence and extinction regimes	Identified conditions for coexistence of robbers, pollinators, and plants; robbers can destabilize mutualism or persist without collapsing the system
[49]	Network-based dynamical model with structural variation	Biodiversity depends on network topology (e.g., nestedness, modularity); interaction strength varies across species; life history traits influence persistence	Network structure varied systematically; interaction matrices parameterized across plausible ecological regimes	Simulations across parameter space; biodiversity measured as fraction of surviving species under different topologies	Found multiple regimes linking nestedness to biodiversity; network structure alone can promote or hinder persistence depending on trait configuration
[50]	Reaction and diffusion model with positive steady-state analysis	Plant and pollinator populations diffuse spatially; mutualism modeled via Beddington–DeAngelis response; steady states represent ecological coexistence	Diffusion coefficients and growth rates selected for analytical tractability; functional response includes saturation and interference terms	Leray–Schauder degree theory used to prove existence of positive steady states; stability explored via monotone dynamical systems	Demonstrated existence and uniqueness of positive steady states; coexistence depends on growth–mortality balance and spatial diffusion rates
[51]	Adaptive dynamics model with trait-mediated trade-offs	Plants evolve interaction traits under nutrient enrichment; traits affect both mutualistic and antagonistic interactions; ecological trade-off is convex	Trait values modulate interaction strength; nutrient enrichment modeled as external forcing; parameters derived from ecological theory	Analytical exploration of evolutionary equilibria; simulations of community assembly under enrichment gradient	Evolution modifies community structure and alleviates priority effects; nutrient enrichment promotes plant diversification into attractive and defensive phenotypes
[52]	Eco-evolutionary network model with trait similarity	Invader success depends on trait similarity to resident species; mutualistic interactions are trait-mediated; network structure influences invasibility	Trait distributions and propagule pressure varied systematically; network metrics (nestedness, modularity) used to define recipient community	Simulations of invasion scenarios; robustness and resilience metrics used to assess impact	Trait dissimilarity enhances invasiveness; network stability better predicts invasibility than topology; multiple introductions increase invasion success
[53]	Age-structured partial differential equation model for unidirectional mutualism	Consumer species structured by age; resource species provides benefit without reciprocal cost; interaction includes both positive and negative effects	Age-dependent interaction kernels, Michaelis-–Menten saturation for resource uptake; parameters derived from biological life history traits	Hopf bifurcation and stability analysis; numerical simulations of periodic solutions	Identified conditions for oscillatory dynamics; age structure induces complex feedbacks; coexistence depends on balance between benefit and exploitation
[54]	Ordinary differential equation system with attractor geometry in mutualistic networks	Bipartite network of plants and pollinators; mutualistic and competitive interactions; attractor architecture governs long-term dynamics	Growth rates and competition and cooperation coefficients defined via network topology; nestedness encoded in interaction matrices	Morse decomposition and Lyapunov function construction; numerical simulations of attractor transitions	Attractor structure determines biodiversity outcomes; nestedness enhances coexistence; topological robustness linked to dynamical stability
[55]	Two-species ordinary differential equation model with behavioral learning	Plants attract pollinators deceptively without offering rewards; pollinators learn to avoid deceptive flowers over time; learning affects visitation rates	Learning encoded via a dynamic cost function; interaction terms modeled with saturating responses; parameters derived from behavioral ecology	Stability and bifurcation analysis; numerical simulations of coexistence and extinction regimes	Learning reduces pollinator visitation to deceptive plants; coexistence depends on deception cost and learning rate; oscillatory dynamics emerge under intermediate conditions
[56]	Delay differential equation model for bumblebee colonies	Bumblebee population structured by colony stages; time delays represent developmental lags; external pressures affect reproduction and survival	Life history parameters derived from Bombus terrestris data; delays calibrated from empirical colony development timelines	Numerical simulations using spline approximations; sensitivity analysis under resource and pesticide stress	Delay structure captures seasonal dynamics and vulnerability; model predicts colony collapse under combined stressors; useful for evaluating conservation strategies
[57]	Genetic hybridization model with Allee effect mitigation	Small populations suffer from pollen limitation and genetic Allee effects; hybridization with co-flowering species improves pollination quality	Single-locus, two-allele genetic model; pollinators modeled as catalytic agents; parameters derived from kinetic reaction theory	Stability analysis of trivial and hybrid equilibria; phase-plane exploration of invasion dynamics	Neutral hybridization removes or reduces Allee thresholds; hybridization facilitates invasion and persistence; implications for conservation and invasion biology
[58]	Fractional-order diffusion model with Lévy flights	Bee-mediated pollen dispersal follows truncated Lévy flight patterns; long-distance dispersal events drive transgene spread; Brownian motion underestimates risk	Dispersal kernels fitted to empirical pollen movement data; fractional diffusion operator used to interpolate between Brownian and Lévy regimes	Numerical solution of fractional PDEs; comparison with classical diffusion predictions; threshold analysis for isolation distances	Lévy-based models predict significantly larger dispersal ranges; isolation distances must be revised upward; model improves risk assessment for GM pollen escape
[59]	Reaction, diffusion, delay system model with Hopf bifurcation	Plant–pollinator system with spatial diffusion and time delay; unidirectional consumer–resource interaction; periodic patterns emerge from instability	Diffusion coefficients and delay terms derived from ecological reasoning; functional responses include saturation and interference	Hopf bifurcation analysis via center manifold and normal form theory; numerical simulations of spatially homogeneous and inhomogeneous solutions	Identified conditions for temporal and spatial oscillations; delay and diffusion jointly drive pattern formation; bifurcation structure predicts ecological transitions
[60]	Trait-based evolutionary model with directional selection	Mutualism evolves via trait matching; regulation of trait variation includes homeostasis, developmental stability, and partner acceptability; selection is directional	Trait space modeled as continuous; mutation bias and environmental noise incorporated; parameters derived from evolutionary theory	Analytical exploration of joint evolution; stability analysis of trait distributions under different regulatory regimes	Developmental stability promotes mutualism evolution; trait regulation affects partner specificity; coevolutionary feedback shapes mutualistic trait architecture
[61]	Hybrid deterministic and agent-based model for oil palm pollination	Pollination by *Elaeidobius* spp. weevils depends on male inflorescence availability; fruit set linked to pollinator dynamics; spatial heterogeneity matters	Deterministic model uses metapopulation dynamics; agent-based model simulates individual weevil behavior; parameters from plantation data	Comparative simulations of both models; fruit set estimates validated against field observations; sensitivity analysis of inflorescence ratios	Agent-based model captures fine-scale dynamics; deterministic model predicts population thresholds; both approaches inform optimal pollinator management for yield improvement
[62]	Adaptive consumer–resource model with trait-mediated facilitation	Two plants compete for pollinators; pollinators adapt preferences based on plant abundance; facilitation and competition co-occur via trait mediation	Functional responses include adaptive foraging; trait distributions influence interaction strength; parameters derived from optimal foraging theory	Isoleg analysis and ideal free distribution framework; numerical simulations of coexistence and exclusion regimes	Adaptive preferences reduce niche overlap and promote coexistence; trait-mediated facilitation alters community structure; coexistence depends on balance between facilitation and competition
[63]	Ordinary differential equation model with behavioral learning and cost-benefit trade-offs	Pollinators learn to avoid deceptive plants; plant population includes rewarding and non-rewarding individuals; learning affects visitation rates	Learning encoded via dynamic feedback; cost-benefit parameters derived from ecological theory and behavioral studies	Hopf bifurcation analysis; numerical simulations of periodic and damped oscillations	Learning induces sustained or damped oscillations; coexistence depends on deception cost and learning rate; behavioral adaptation stabilizes mutualism
[64]	Three-species dynamical system with competition, parasitism, and mutualism	Plants interact with pollinators (mutualism) and nectar robbers (parasitism); pollinators and robbers compete indirectly for floral resources	Functional responses include Beddington-–DeAngelis and Holling type II; parameters derived from the ecological literature and invasion theory	Global stability and persistence analysis; bifurcation diagrams of coexistence regimes	Coexistence possible under intermediate parasitism and competition; mutualism can persist despite robbers; extinction thresholds depend on interaction efficiency
[65]	Dimension-reduced model of mutualistic network collapse	High-dimensional mutualistic networks exhibit tipping points; dimension reduction captures essential dynamics; stochastic perturbations affect resilience	Reduction to 2D system using weighted averaging of empirical networks; parameters derived from 59 real-world datasets	Comparison of reduced model predictions with full network simulations; robustness tested under structural perturbations	Reduced model accurately predicts tipping points; resilience depends on network structure and interaction strength; framework generalizable to other complex systems
[66]	Compartmental population model for pyrophite shrub dynamics	Ulex parviflorus population structured by age and reproductive status; fire regimes influence biomass and regeneration; Mediterranean ecosystem context	Growth, flowering, and seed dispersal modeled via nonlinear differential equations; parameters calibrated from field data in Castellón, Spain	Numerical simulations of post-fire recovery and reproductive cycles; sensitivity analysis of biomass and seed bank dynamics	Pyrophite shrubs exhibit structured recovery under fire disturbance; reproductive success depends on spatial distribution and ecological thresholds
[67]	Three-species nonlinear ordinary differential equation model with limit cycle emergence	Plant–pollinator mutualism coupled with herbivory; herbivores reduce plant biomass and indirectly affect pollinator visitation; functional responses are nonlinear	Type IV functional responses for herbivory and mutualism; parameters selected for dynamical richness and ecological realism	Hopf-–Andronov bifurcation theorem applied; Lyapunov coefficient used to confirm stability of limit cycle; numerical simulations support analytical results	Demonstrated existence of a stable limit cycle; herbivory can destabilize mutualism and induce oscillatory dynamics; coexistence depends on interaction strength and saturation effects
[68]	Eco-evolutionary simulation model with genetic algorithm	Obligate pollination mutualism; plant and pollinator traits evolve under fitness trade-offs; genetic algorithm simulates adaptive dynamics	Heuristic Lotka–Volterra-type model; fitness landscapes and trait distributions encoded in algorithm; parameters varied across simulations	Zero-isocline analysis and trait distribution mapping; robustness tested across multiple evolutionary runs	Trade-offs between cost and benefit shape mutualist niches; genetic algorithm reveals multiple stable eco-evolutionary regimes; obligate mutualism can persist under constrained trait evolution
[69]	Network-based model with exploitative competition	Pollinators compete for shared plant resources; exploitative competition affects network topology and species abundance; mutualistic links evolve adaptively	Interaction matrix constructed from rewiring rules; competition strength varied systematically; parameters derived from network theory	Analytical inversion of interaction matrix; simulations of network evolution under competition pressure	Exploitative competition increases plant abundance; pollinator hubs emerge asymmetrically; network rewiring enhances mutualistic benefit while minimizing competition cost
[70]	Two-patch ordinary differential equation model with dispersal and mutualism	Pollinators and plants interact in two spatial patches; dispersal affects persistence and abundance; mutualism is patch-dependent	Resource-service exchange modeled via nonlinear terms; dispersal rates and survival thresholds calibrated from theoretical ecology	Stability analysis of equilibria; numerical simulations of dispersal scenarios and population trajectories	Dispersal enhances pollinator abundance even under low plant density; patch quality influences mutualistic outcomes; small dispersal can outperform non-dispersal in both persistence and productivity
[71]	Three-species ordinary differential equation model with intermediary nectar dynamics	Nectar acts as intermediary resource between plant and pollinator; nectar dynamics influence mutualism persistence; nectar rapidly reaches quasi-steady state in reduced model	Nectar decay, production, and consumption rates derived from the ecological literature; reduced model assumes fast nectar dynamics	Analytical comparison of full and reduced models; bifurcation analysis and numerical simulations	Initial nectar density critically affects pollinator survival; reduced model captures long-term dynamics; nectar-mediated feedback shapes coexistence thresholds
[72]	Conceptual synthesis of mutualistic network theory	Network structure and species traits jointly determine mutualism dynamics; adaptive foraging and trait matching improve predictive capacity	Parameters drawn from empirical datasets and theoretical models; emphasis on trait-based and mechanistic approaches	Comparative review of modeling frameworks; integration of empirical validation strategies	Advocates for biologically grounded models; trait-based and adaptive mechanisms enhance prediction of network responses to perturbations
[73]	Two-plant, one-animal model with adaptive foraging	Animal mediator (mutualist or exploiter) adapts foraging preferences; plant coexistence mediated by behavioral feedback; animal density fixed	Preferences evolve to maximize fitness; generalized isocline framework used; competition strength varied systematically	Differential inclusion and sliding mode analysis; numerical simulations of coexistence regimes	Exploiter generalism promotes coexistence under strong competition; mutualist specialization yields alternative stable states; adaptive behavior reshapes competitive outcomes
[74]	Three-species ordinary differential equation model with food and toxin production	Plant produces both nectar (mutualism) and toxin (defense); pollinator and herbivore interact with plant via distinct pathways; trade-offs govern coexistence	Functional responses include saturation and inhibition; toxin production modeled as dynamic trait; parameters derived from ecological experiment	Hopf bifurcation and persistence analysis; numerical simulations of oscillatory and steady-state regimes	Toxin production modulates herbivore suppression and pollinator survival; coexistence possible via intermediate defense levels; excessive defense leads to collapse
[75]	Three-species ordinary differential equation model with pesticide-induced mortality	Pesticides reduce pollinator survival and indirectly affect plant reproduction; mutualism depends on energetic reward; extinction thresholds exist	Mortality rates and reward thresholds derived from ecological theory; pesticide effects modeled as additive mortality terms	Stability analysis of equilibria; numerical simulations under varying pesticide intensities	High energetic reward can buffer pesticide impact; low reward leads to plant extinction; mutual dependence is sensitive to pesticide pressure
[76]	Ordinary differential equation model with nonlinear mutualism and pesticide feedback	Pollinator mortality increases with pesticide exposure; mutualism modeled via Beddington-–DeAngelis response; plant growth depends on pollination service	Pesticide toxicity modeled as a dynamic variable; parameters derived from empirical studies and theoretical ecology	Global stability and persistence analysis; bifurcation diagrams of extinction and coexistence regimes	Mutualism persists under moderate pesticide levels; excessive toxicity leads to collapse; coexistence thresholds depend on pollinator resilience and plant reward rates
[77]	Replicator and ordinary differential equation hybrid model with floral deception	Plants produce nectar-rich or nectarless flowers; pollinators adopt selective or non-selective foraging strategies; evolutionary game dynamics shape population structure	Nectar cost and cheater efficiency encoded in replicator equations; interaction terms derived from behavioral ecology	Bifurcation analysis of periodic and steady-state regimes; numerical simulations of invasion and persistence scenarios	Nectarless flowers can persist via cyclic dynamics; pollinator learning affects strategy evolution; coexistence depends on cost-benefit asymmetry and foraging discrimination
[78]	Three-species ordinary differential equation model with intermediary nectar dynamics	Nectar acts as intermediary resource between plant and pollinator; nectar dynamics influence persistence; plant cannot survive without pollination	Nectar decay, production, and consumption rates derived from ecological experiments; reduced model assumes fast nectar equilibrium	Analytical comparison of full and reduced models; global dynamics and persistence conditions derived rigorously	Initial nectar density determines persistence; low decay rates favor coexistence; intermediary resource mediates survival thresholds and system resilience
[79]	Nonlinear ordinary differential equation model with temperature-dependent mortality	Rising environmental temperature increases pollinator mortality and reduces plant reproduction; mutualism is sensitive to thermal stress	Temperature effects modeled via exponential mortality terms; ecological parameters derived from the climate and pollination literature	Local and global stability analysis; numerical simulations under varying temperature regimes	Elevated temperature reduces pollinator persistence and plant biomass; mutualism collapses under extreme warming; mitigation requires cooling interventions
[80]	Network-based co-adaptation model with dynamic link weights	Mutualistic networks adapt both structurally and dynamically; co-adaptation enhances resilience under perturbations; link weights evolve with species abundance	Link weights updated via feedback rules; empirical networks used for calibration; heterogeneity and connectance preserved	Comparative simulations of static, adaptive, and co-adaptive models; robustness tested under species loss scenarios	Co-adaptation increases resilience without altering connectance; dynamic feedback buffers against coextinction; model generalizable to other complex systems
[81]	Three-species ordinary differential equation model with antagonism between mutualists	Two mutualists share a partner species but interact antagonistically; antagonism may be consumptive or non-consumptive; life stages explicitly modeled	Interaction strengths and specialization levels varied systematically; antagonism encoded as direct negative feedback	Stability and persistence analysis; bifurcation exploration of oscillatory and extinction regimes	Antagonism reduces mutualist persistence; indirect effects dominate at high antagonism rates; specialization modulates system resilience
[82]	Empirical network analysis with epidemiological modeling	Landscape simplification alters plant-pollinator network structure; pathogen prevalence shaped by diet breadth and connectance; dilution effect emerges in complex networks	Eleven empirical networks analyzed; pathogen prevalence measured via molecular assays; network metrics computed from field data	Structural equation modeling and simulation of disease dynamics; robustness tested across landscape gradients	Simplified landscapes increase pathogen prevalence; higher connectance reduces outbreak risk; dominant species’ diet breadth mediates community-level infection patterns
[83]	Delay differential equation model with empirical calibration	Fruit yield in dioecious crops depends on orchard layout, flower sex ratio, and pollinator density; pollinator behavior has diminishing returns at high density	Empirical data from kiwifruit orchards in New Zealand; Latin hypercube sampling used for sensitivity analysis	Simulations of fruit set under varying orchard configurations; model validated against field observations	Plant traits and layout more influential than pollinator density; optimal yield achieved with 65–75% female flowers and ≥6 bees per 1000 flowers
[84]	Fractional-order differential model with Atangana-–Baleanu derivative	Nectar acts as intermediary resource; fractional calculus captures memory effects and non-locality in pollination dynamics	Fractional order α∈(0,1) varied systematically; stability analyzed via Picard-–Lindelöf method	Numerical simulations using Adams–Bashforth scheme; stability tested across fractional orders	Fractional models outperform classical ODEs in capturing system memory; persistence depends on nectar dynamics and fractional order
[85]	Mechanistic transmission model with empirical viral assays	Deformed wing virus (DWV) transmits between bee species via shared flowers; transmission is bidirectional and density-dependent	Laboratory experiments with *Apis mellifera* and *Bombus impatiens*; viral load quantified via molecular assays	Mathematical simulations of transmission dynamics; dilution effect tested via floral abundance scenarios	DWV spreads via shared floral resources; increasing floral abundance reduces transmission; managing *Varroa* mites in honeybees mitigates spillover
[86]	Ordinary differential equation model with adaptive foraging by herbivores and pollinators	Two plants compete for shared mutualists and exploiters; animal preferences adapt to plant density; indirect interactions shape coexistence	Trait-mediated interactions modeled via isolegs and ideal free distribution; parameters varied across consumer abundance	Stability analysis of alternative states; simulations of coexistence under adaptive behavior	Adaptive preferences promote coexistence at high consumer abundance; low abundance leads to specialization and exclusion; insect decline alters plant community structure
[87]	Adaptive evolutionary model of nectar provisioning traits	Plants evolve nectar traits (production rate and reservoir volume) under selective pressures from pollinator interactions; traits co-evolve as a suite	Cost-benefit trade-offs modeled explicitly; ecological constraints include pollinator conversion efficiency and plant productivity	Analytical exploration of evolutionary equilibria; simulations of trait dynamics under varying ecological conditions	Higher nectar provisioning evolves under pollinator limitation, compensatory investment across traits stabilizes mutualism; indirect selection shapes trait architecture
[88]	Stochastic–deterministic hybrid model for hoverfly pollination control	Tomato flowers lack nectar; hoverfly density must be supplemented via feeding; pollination success depends on adult density and feeding strategy	Stochastic model estimates required hoverfly density; deterministic optimal control model minimizes feeding cost; parameters derived from greenhouse crop data	Simulation of pollinator dynamics and fruit yield; theoretical validation of control strategy under economic constraints	Supplementary feeding maintains economically viable hoverfly density; optimal control reduces cost; model supports hoverfly-based pollination in nectar-deficient crops
[89]	Epidemiological synthesis with genotype replacement modeling	DWV-A and DWV-B genotypes co-circulate in honeybee populations; DWV-B exhibits higher transmission and virulence; genotype interference affects prevalence	Global dataset (2008–2021) analyzed; mathematical model incorporates genotype competition and host co-infection dynamics	Empirical prevalence data from Germany, Italy, and the UK; model predictions compared to observed genotype shifts	DWV-B is replacing DWV-A globally; genotype interference drives replacement; implications for wild pollinators and beekeeping practices
[90]	Dose–response transmission model with pathogen transport	Pathogen transport via mechanical vectors (e.g., pollinators) alters exposure distribution; transmission depends on host dose–response curve	Two transmission scenarios modeled: amplification and dilution; dose–response functions derived from empirical infection thresholds	Analytical derivation of infection risk under varying transport regimes; simulations of disease spread in pollinator networks	Transport amplifies or dilutes transmission depending on dose–response shape; oversimplified models misestimate risk; framework improves epidemiological predictions
[91]	Consumer–resource ordinary differential equation models with reproductive benefit mechanisms	Plant reproductive benefits arise via pollination or seed dispersal; benefits affect seed set, germination, or recruitment; mutualism may be obligate or facultative	Foraging rate functions and benefit pathways modeled explicitly; parameters varied across ecological scenarios	Stability and bifurcation analysis; simulations of low-density thresholds and Allee effects	Pollination and seed dispersal mutualisms exhibit distinct dynamics; bistability and collapse thresholds depend on benefit mechanism and partner density
[92]	Network-based resilience analysis with dimension reduction	Hybrid ecological networks include mutualism, herbivory, and antagonism; resilience assessed via species contribution and extinction vulnerability	Interaction matrices constructed from hybrid network topology; resilience evaluated via reduced-dimensional metrics	Simulation of species removal and perturbation scenarios; resilience mapped across species categories	Strong contributors to resilience are more extinction-prone; plants are most vulnerable; network structure influences robustness under perturbation
[93]	Non-autonomous nonlinear ordinary differential equation model with seasonal forcing and parasitism	Honey bee population dynamics influenced by seasonal egg-laying and parasitism (e.g., *Varroa* mites); seasonality modulates colony resilience	Seasonality encoded via time-dependent birth rates; parasitism modeled as density-dependent mortality; parameters derived from empirical data	Hopf bifurcation analysis; simulations of collapse and recovery under seasonal and parasitic stress	Seasonality can stabilize or destabilize colonies depending on timing; parasitism induces collapse via bifurcation; synergistic effects shape colony survival
[94]	Delay differential equation model with dual time lags	Plant–pollinator interactions subject to two distinct delays (e.g., response and maturation); delays influence stability and oscillatory behavior	Characteristic equations derived from delay structure; delays treated as bifurcation parameters; ecological rates assumed to be constant	Center manifold and normal form theory applied; numerical simulations of periodic solutions and stability regions	Multiple delays induce Hopf bifurcations and periodic dynamics; delay asymmetry affects system persistence; explicit conditions derived for oscillation onset
[95]	Analytical framework for structural stability in mutualistic–competitive networks	Mutualistic networks include interspecific competition among plants and pollinators; structural stability defined as coexistence feasibility under perturbation	Total of 50 empirical networks used; competition encoded via weighted adjacency matrices; mutualism modeled with saturating responses	Analytical derivation of feasibility domains; numerical simulations of stability under network rewiring	Competitive structure strongly influences coexistence; mutualism alone insufficient for stability; new metric links network architecture to resilience thresholds
[96]	Eco-evolutionary model with adaptive disinvestment	One-sided population decline triggers adaptive reduction in mutualistic investment; feedback loops can accelerate collapse or stabilize interaction	Trade-off between independent growth and mutualistic investment modeled as concave function; adaptation rates varied systematically	Simulations of co-evolutionary trajectories; bifurcation analysis of collapse and recovery regimes	Disinvestment by undisturbed partner precedes collapse; slow adaptation or high initial investment delays extinction; co-evolution essential for recovery
[97]	Socio-mutualistic network model with optimal conservation strategy	Pollinator dynamics coupled with human conservation norms; tipping points emerge from structural and behavioral feedbacks	Network topology varied across nestedness levels; conservation norms applied selectively to pollinator nodes	Dynamical analysis of reduced model; simulations across empirical and synthetic networks	Optimal conservation strategy prevents collapse with minimal intervention; intermediate nestedness most responsive; social norms amplify resilience
[98]	Generalized stressor model for hive and forager bees	Stressors affect bees via transmissibility, lethality, impairment, and timing; colony collapse emerges from labor destabilization and precocious foraging	Stressor attributes generalized across multiple dimensions; model integrates prior CCD frameworks; parameters derived from empirical studies	Validated against emergent colony behaviors; simulations of collapse thresholds under stressor variation	Sublethal stressors sufficient to trigger collapse; timing and impairment level critical; model supports holistic stressor management in conservation
[99]	Ordinary differential equation based community model with antagonism–mutualism continuum	Herbivores reduce pollinator visitation via indirect limitation; mutualistic and antagonistic interactions co-occur; stability assessed across interaction gradients	Interaction strengths varied systematically; network architecture encoded via adjacency matrices; parameters derived from ecological literature	Temporal and compositional stability analyzed; simulations of species persistence and network robustness	Pollinator limitation enhances both temporal and compositional stability; herbivory indirectly promotes persistence; network architecture–stability relationship shifts under limitation
[100]	Behaviorally modified predator–prey–mutualism model	Predation on pollinators alters foraging behavior; behavioral avoidance stabilizes mutualism; direct predation alone insufficient for stability	Pollinator behavior modeled via adaptive response functions; predation rates and avoidance thresholds varied across scenarios	Stability analysis of modified mutualism; simulations of predator-induced behavioral shift	Behavioral modification stabilizes mutualism under predation; avoidance behavior critical for persistence; predator pressure reshapes interaction dynamics
[101]	Network-based thermal response model with tipping point detection	Climate warming alters species-level physiological traits; mutualistic networks exhibit tipping points under thermal stress; generalists play stabilizing roles	Total of 139 empirical networks analyzed; thermal sensitivity encoded via trait-dependent growth and mortality rates	Reduced two-dimensional model used for bifurcation analysis; simulations across temperature gradients	Rising temperature induces rapid transitions in low-strength networks; generalists delay collapse; network structure and thermal traits jointly determine resilience
[102]	Partial differential equation system with chemotaxis-enhanced reaction rates	Chemotaxis enhances biological reaction efficiency; organisms move toward chemical gradients; surface chemotaxis considered for biological realism	Chemotactic flux modeled via Keller–Segel-type terms; reaction rates compared with pure diffusion; parameters derived from biological signaling contexts	Analytical estimates of convergence rates; sharp inequalities derived for Fokker–Planck operators	Chemotaxis significantly enhances reaction success; convergence to equilibrium accelerated; framework applicable to immune signaling, reproduction, and pollination
[103]	Trait-based evolutionary model with trophic structure	Plants and pollinators co-evolve under mutualistic and competitive pressures; trait polymorphisms emerge via Darwinian dynamics; niche breadth influences diversification	Trait matching and competition encoded via continuous trait space; ecological rates derived from trophic interactions	Analytical derivation of evolutionary branching conditions; simulations of polymorphism emergence	Broad plant niches promote phenotypic diversification; mutualistic generalism triggers cascading trait divergence; co-diversification driven by bottom-up control
[104]	Delay differential equation model with brood mortality	Brood deaths induced by insecticide exposure affect colony dynamics; time delay represents developmental lag; collapse thresholds explored	Mortality and delay parameters derived from empirical bee life cycle data; insecticide effects modeled as external forcing	Stability and sensitivity analysis of equilibria; numerical simulations with real data	Brood mortality significantly reduces colony resilience; no Hopf bifurcation within biologically plausible delay range; model supports policy design for insecticide regulation
[105]	Stochastic branching process model with behavioral resistance	Grooming and hygienic behaviors reduce DWV outbreak probability in *Varroa*-infested colonies; transmission occurs via vector and contact routes	Transmission probabilities and behavioral efficacy derived from empirical studies; branching process theory applied to outbreak dynamics	Analytical computation of outbreak probabilities; simulations across behavioral scenarios	Hygienic behavior most effective at reducing DWV outbreaks; grooming reduces vector transmission to near zero; behavioral selection enhances colony survival
[106]	Nonlinear dynamical system with bifurcation analysis in parameter space	Honeybee—mite interactions exhibit multistability, chaos, and bifurcation structures; parameter variation reveals complex attractor landscapes	Interaction rates and mortality parameters varied systematically; bifurcation parameters mapped across biologically relevant ranges	Numerical exploration of phase space; identification of Arnold tongues, jellyfish structures, and saddle regions	System exhibits rich dynamical regimes including chaos and multistability; parameter tuning critical for colony persistence; visual structures aid in ecological interpretation
[107]	Three-species nonlinear ordinary differential equation model with limit cycle dynamics	Pollinators and plants form a mutualistic pair; herbivores consume plants and indirectly reduce pollinator visitation; functional responses are of type IV	Saciety and saturation effects encoded via nonlinear terms; ecological rates selected for dynamical richness	Hopf–Andronov bifurcation theorem applied; Lyapunov coefficient used to confirm stability of limit cycle; numerical simulations support analytical results	Herbivory destabilizes mutualism and induces oscillatory dynamics; coexistence depends on interaction strength and saturation thresholds
[108]	Consumer–resource network model with adaptive foraging	Floral resource availability constrains pollinator specialization; network structure emerges from adaptive foraging and resource depletion	Lotka–Volterra framework with dynamic floral resource pool; parameters varied across resource and animal density gradients	Generalized linear models used to explain nestedness, modularity, and specialization; simulations across 3812 networks	Low floral resource availability reduces specialization; resource dynamics distinct from plant density; adaptive foraging mediates network structure
[109]	Adaptive dynamics model of floral handedness evolution	Mirror-image flowers evolve from mixed to fixed handedness; stylar orientation affects pollination efficiency and mating patterns; inbreeding depression penalizes geitonogamy	Trait evolution modeled via adaptive dynamics; genetic architecture assumed plausible; ecological parameters include pollinator efficiency and plant density	Stability analysis of dimorphic vs. monomorphic states; population genetics simulations validate transitions	Dimorphic enantiostyly evolves under moderate inbreeding and high pollination efficiency; ecological shifts may reverse dimorphism; model supports convergence in floral asymmetry
[110]	Population dynamics model of lethal deceptive pollination	Plants trap and kill pollinators without reward; coexistence depends on vegetative reproduction and sex transition rates; disturbances affect demographic stability	Empirical data from *Arisaema* species used to calibrate model; disturbance scenarios include deer abundance, habitat loss, and plant theft	Stability analysis and extinction thresholds explored; simulations under multiple disturbance regimes	Lethal deception maintained under high vegetative reproduction and slow sex transition; co-extinction risk elevated under strong attraction and anthropogenic disturbance
[111]	Impulsive reaction–diffusion model on a periodically evolving domain	Plant–pollinator system subject to periodic habitat changes and impulsive disturbances; domain evolution affects dispersal and persistence	Ecological reproduction index defined; impulsive effects modeled via discontinuous terms; domain evolution rate treated as bifurcation parameter	Upper–lower solution method applied; numerical simulations of extinction and persistence scenarios	High domain evolution rate promotes pollinator survival; impulsive effects can override spatial benefits; coexistence depends on timing and magnitude of impulses
[112]	Stochastic partial differential equation model with degenerate diffusion	Plant–pollinator dynamics influenced by environmental noise; degenerate diffusion captures spatial heterogeneity and dispersal limitations	Diffusion coefficients vary with population density; stochastic terms modeled via Brownian motion; permanence conditions derived analytically	Stochastic comparison principle and Lyapunov function used; extinction and persistence regions identified	Degenerate diffusion enhances extinction risk under low density; stochasticity can stabilize or destabilize dynamics; permanence requires bounded noise intensity
[113]	Mechanistic ordinary differential equation model with separate pollen and nectar dynamics	Pollinators consume nectar and transfer pollen independently; interspecific pollen transfer reduces pollination efficiency; mutualism may shift to antagonism	Pollen and nectar modeled as distinct resources; pollinator efficiency and interference encoded via saturating functions	Analytical derivation of pollination efficiency; simulations of mutualism-antagonism transitions	Separation of pollen and nectar dynamics reveals hidden antagonism; interspecific pollen transfer reduces plant fitness; ecological context determines net interaction outcome
[115]	Nonlinear dynamical system with predation on pollinators	Predators consume pollinators and destabilize mutualism; plant—pollinator interaction saturates at high density; predator–pollinator dynamics coupled	Saturated mutualism modeled via Holling-type functions; predation rate and initial density varied across simulations	Stability and bifurcation analysis; numerical exploration of transcritical and Hopf bifurcations	High predator density leads to pollinator extinction; mutualistic strength buffers predation impact; coexistence possible under low predation and strong mutualism
[114]	Tripartite ordinary differential equation model with facultative and obligate mutualism and parasitism	Plant–pollinator–parasite system; pollinators may be facultative or obligate; parasites affect pollinator fitness and system stability	Facultative and obligate behavior encoded via growth and mortality terms; bifurcation parameters include parasite conversion and death rates	Bifurcation analysis of subsystems and full model; numerical simulations of multistability and oscillatory regimes	Facultative pollinators enhance resilience via multistability; obligate systems prone to collapse; novel bifurcation patterns (e.g., wavebow) characterize amplitude transitions
[116]	Delayed reaction, diffusion model with memory-based diffusion	Pollinator movement influenced by spatial memory; delay affects diffusion and pattern formation; periodicity emerges from delay–diffusion interplay	Memory delay incorporated via modified Fick’s law; diffusion coefficients and delay terms varied systematically	Hopf bifurcation and Lyapunov–Schmidt reduction applied; simulations of spatial and temporal periodic solution	Memory-based diffusion induces spatial heterogeneity and oscillations; delay triggers stability switch; model captures biologically realistic movement biases

**Table 5:** Summary of retained studies according to biological topics and other characteristics.

Modeling Domain	Assumptions	Parametrization	Validation	Findings	Policy	Works
Biological consistency and population viability	Non-negative, bounded population variables.	Logistic or saturating growth; constrained initial conditions.	Analytical consistency.	Avoids spurious extinction or explosion.	Supports viability thresholds.	[15,19,20]
Long-term dynamics and species persistence	Equilibrium-based persistence or extinction.	Reproduction, mortality, and interaction sensitivity.	Stability via Lyapunov function and linearization.	Identifies resilience thresholds.	Informs sustainability planning.	[10,11,12]
Regime shifts and critical transitions	Threshold-driven qualitative change.	Bifurcation parameters.	Bifurcation diagrams.	Reveals tipping points.	Enables adaptive management.	[16,30,53]
Mutualism and network structure	Mutualistic coexistence and spatial structure.	Patch connectivity and trait evolution.	Network simulations.	Biodiversity maintenance.	Corridor design and zoning.	[13,16,18]
Seasonal and oscillatory behavior	Seasonal forcing and delay effects.	Time-dependent coefficients.	Periodicity analysis.	Captures seasonal fluctuations.	Supports crop planning.	[30,47,65]
Simulation and empirical calibration	Empirical realism.	Optimization and sensitivity analysis.	Simulation observed data.	Enhances credibility.	Enables data-driven decisions.	[19,25]
Intervention and optimization	Ecological outcomes modifiable via control.	Pontryagin’s maximum principle and dynamic programming.	Optimality conditions.	Cost-effective strategies.	Informs adaptive conservation.	[88,97]

## Data Availability

The original contributions presented in this study are included in the article.

## Appendix A. Details on Counting the Regions

In order to count the regions, we considered the affiliations declared by the authors. For instance, in [10], the authors, Soberon and Rio, declared Mexico as their affiliation, and in [11], the author, Wells, declared USA for the affiliation. Extensive details for the retained list are presented on Table A1. We note that in the case of the same author declaring multiple affiliations, we considered only the first declared region.

**Table A1:** Affiliations of the authors declared on the retained list. Here, RR stands for retained reference.

RR	Affiliations	RR	Affiliations	RR	Affiliations	RR	Affiliations
[10]	Mexico (2)	[37]	USA (2), China (2)	[64]	China (1)	[91]	USA (4)
[11]	USA (1)	[38]	Australia (3)	[65]	USA (4), China (2), UK (1)	[92]	China (3)
[12]	USA (1)	[39]	Netherlands (2), France (1)	[66]	Spain (4)	[93]	USA (6)
[13]	USA (2)	[40]	Ecuador (1), Spain (2)	[67]	Mexico (2)	[94]	China (4)
[14]	Sweden (2)	[41]	USA (5), Brazil (1)	[68]	Australia (1), UK (1)	[95]	China (1), Brazil (1), Netherlands (1), France (1), Spain (4)
[15]	USA (1)	[42]	Mexico (3)	[69]	R. of Korea (3)	[96]	Germany (4)
[16]	Italy (1)	[43]	Mexico (1)	[70]	China (1)	[97]	India (3)
[17]	UK (3)	[44]	China (2)	[71]	China (1)	[98]	USA (2)
[18]	USA (2)	[45]	China (2)	[72]	USA (1)	[99]	China (2)
[19]	Mexico (2)	[46]	China (1), USA (2)	[73]	Czech Republic (2)	[100]	Japan (2)
[20]	Argentina (2)	[47]	Mexico (2)	[74]	China (3)	[101]	India (3)
[21]	Chile (5)	[48]	China (3)	[75]	India (2)	[102]	USA (4)
[22]	Israel (2)	[49]	USA (4)	[76]	China (2), USA (1)	[103]	Czech Republic (3)
[23]	UK (3), Canada (1), USA (1)	[50]	China (3)	[77]	China (3)	[104]	Bulgaria (3)
[24]	Germany (2)	[51]	France (2)	[78]	China (4)	[105]	South African (2), Netherlands (1)
[25]	Argentina (2), Germany (1)	[52]	South African (2)	[79]	India (2)	[106]	India (2)
[26]	Mexico (2)	[53]	China (1), France (1), USA (1)	[80]	China (3), USA (2)	[107]	Mexico (3)
[27]	Canada (3)	[54]	Ecuador (1), Spain (2)	[81]	USA (2), France (1), Switzerland (1)	[108]	South Africa (4)
[28]	China (1), USA (3)	[55]	Mexico (2)	[82]	USA (6), UK (1)	[109]	Netherlands (2), Canada (1)
[29]	USA (4)	[56]	USA (5), Sweden (2)	[83]	USA (6), New Zeland (6)	[110]	Japan (3)
[30]	China (3)	[57]	Canada (2), Australia (2)	[84]	Saudi Arabia (2), Mexico (1), Pakistan (1)	[111]	China (4)
[31]	USA (1)	[58]	Belgium (2), Canada (2), Netherlands (1)	[85]	USA (6)	[112]	China (2)
[32]	USA (3)	[59]	China (2), USA (1)	[86]	Czech Republic (3)	[113]	Czech Republic (1)
[33]	China (2)	[60]	Japan (1)	[87]	USA (3)	[115]	China (1), Canada (1)
[34]	China (1)	[61]	South Africa (1), France (2)	[88]	Spain (2), Hungary (6)	[114]	India (3)
[35]	Chile (3), USA (1)	[62]	Czech Republic (2)	[89]	Italy (4), Germany (7)	[116]	Czech Republic (1)
[36]	UK (5)	[63]	Mexico (2)	[90]	USA (4)		

## Appendix B. Details on Journals for Retained List

We identified the journals and searched for the impact factor, H index and quartiles, which are presented in Table A2.

**Table A2:** List of the journals appearing in the retained reference list.

Journal	H Index	SJR	Quartile
*Agronomy-Basel*	114	3.7	Q1
*Alexandria Engineering Journal*	112	5.6	Q1
*American Naturalist*	236	3	Q2
*Annals of Botany*	215	4.1	Q1
*Applied Ecology and Environmental Research*	48	0.9	Q4
*Applied Mathematical Modelling. Simulation and Computation for Engineering and Environmental Systems*	150	4.2	Q1
*Applied Mathematics and Computation*	182	3.1	Q1
*Applied Sciences-Basel*	162	2.7	Q2
*Biosystems*	85	0.392	Q2
*Boletín de la Sociedad Matemática Mexicana. Third Series*	20	0.414	Q2
*Bulletin of Mathematical Biology*	101	0.702	Q1
*Chaos, Solitons & Fractals*	175	1.184	Q1
*Chaos. An Interdisciplinary Journal of Nonlinear Science*	–	–	–
*Communications in Nonlinear Science and Numerical Simulation*	143	0.956	Q1
*Discrete and Continuous Dynamical Systems. Series A*	80	1.065	Q1
*Discrete and Continuous Dynamical Systems. Series B. A Journal Bridging Mathematics and Sciences*	65	0.735	Q1
*Discrete and Continuous Dynamical Systems. Series S*	43	0.514	Q2
*Ecological Modelling*	189	0.896	Q1
*Ecological Research*	87	0.616	Q2
*Ecology*	345	5.5	Q1
*Ecology and Evolution*	109	0.858	Q1
*Ecology Letters*	330	9.8	Q1
*European Journal of Applied Mathematics*	53	0.750	Q2
*Evolution*	227	3.4	Q2
*Evolutionary Applications*	95	1.362	q1
*Evolutionary Ecology*	96	0.645	Q2
*Evolutionary Ecology Research*	82	–	–
*International Journal for Parasitology-Parasites and Wildlife*	44	0.618	Q1
*International Journal of Bifurcation and Chaos in Applied Sciences and Engineering*	120	0.596	Q1
*International Journal of Biomathematics*	38	0.527	Q2
*Journal of Applied Ecology*	216	6.2	Q1
*Journal of Biological Dynamics*	46	0.597	Q2
*Journal of Biological Systems*	39	0.487	Q2
*Journal of Ecology*	219	6.1	Q1
*Journal of Evolutionary Biology*	148	0.921	Q1
*Journal of Mathematical Biology*	111	0.921	Q1
*Journal of Mathematics*	30	0.322	Q3
*Journal of Statistical Mechanics: Theory and Experiment*	95	0.373	Q3
*Journal of the European Mathematical Society*	68	3.043	Q1
*Journal of the Royal Society Interface*	177	1.025	Q1
*Journal of Theoretical Biology*	178	0.532	Q2
*Lobachevskii Journal of Mathematics*	31	0.435	Q2
*Mathematical Biosciences*	114	0.555	Q2
*Mathematical Methods in the Applied Sciences*	87	1.991	Q1
*Modeling Earth Systems and Environment*	66	0.654	Q1
*Natural Resource Modeling*	38	0.521	Q2
*Nonlinear Analysis. Real World Applications. An International Multidisciplinary Journal*	106	1.168	Q1
*Nonlinear Studies. The International Journal*	22	0.229	Q4
*Oikos*	210	1.438	Q1
*Physica A. Statistical Mechanics and its Applications*	195	0.669	Q2
*Physica D: Nonlinear Phenomena*	154	0.940	Q1
*PLoS ONE*	467	3.3	Q1
*PLoS Pathogens*	260	5.5	Q1
*Proceedings of the National Academy of Sciences of the United States of America*	896	10.8	Q1
*Royal Society Open Science*	92	0.795	Q1
*Scientific Reports*	347	4.3	Q1
*Theoretical Ecology*	45	0.524	Q2
*Theoretical Population Biology*	99	0.563	Q2
